# Dynamic nanoscale morphology of the ER surveyed by STED microscopy

**DOI:** 10.1083/jcb.201809107

**Published:** 2019-01-07

**Authors:** Lena K. Schroeder, Andrew E.S. Barentine, Holly Merta, Sarah Schweighofer, Yongdeng Zhang, David Baddeley, Joerg Bewersdorf, Shirin Bahmanyar

**Affiliations:** 1 Department of Cell Biology, School of Medicine, Yale University, New Haven, CT; 2 Department of Biomedical Engineering, Yale University, New Haven, CT; 3 Department of Molecular Cellular and Developmental Biology, Yale University, New Haven, CT; 4 Nanobiology Institute, Yale University, West Haven, CT; 5 Auckland Bioengineering Institute, University of Auckland, Auckland, New Zealand

## Abstract

The endoplasmic reticulum (ER) is composed of interconnected membrane sheets and tubules. Superresolution microscopy recently revealed densely packed, rapidly moving ER tubules mistaken for sheets by conventional light microscopy, highlighting the importance of revisiting classical views of ER structure with high spatiotemporal resolution in living cells. In this study, we use live-cell stimulated emission depletion (STED) microscopy to survey the architecture of the ER at 50-nm resolution. We determine the nanoscale dimensions of ER tubules and sheets for the first time in living cells. We demonstrate that ER sheets contain highly dynamic, subdiffraction-sized holes, which we call nanoholes, that coexist with uniform sheet regions. Reticulon family members localize to curved edges of holes within sheets and are required for their formation. The luminal tether Climp63 and microtubule cytoskeleton modulate their nanoscale dynamics and organization. Thus, by providing the first quantitative analysis of ER membrane structure and dynamics at the nanoscale, our work reveals that the ER in living cells is not limited to uniform sheets and tubules; instead, we suggest the ER contains a continuum of membrane structures that includes dynamic nanoholes in sheets as well as clustered tubules.

## Abstract (precis)

Schroeder et al. quantitatively evaluate ER architecture in live cells at a ∼50-nm resolution through stimulated emission depletion (STED) microscopy. The ER is not limited to uniform sheets and tubules; they observe dynamic, nanoscale-size holes in ER sheets termed “nanoholes,” and they characterize the effects of perturbations of reticulons, Climp63, and the microtubule cytoskeleton on ER membrane nanostructures.

## Introduction

The ER is the largest membrane-bound organelle in eukaryotic cells. ER membranes are spread throughout the cytoplasm to perform essential functions in protein and lipid synthesis as well as calcium signaling. The continuous membranes of the ER extend from the nuclear envelope (NE) as stacks of sheets and transition into a network of tubules and sheets at the cell periphery (Baumann and Walz, 2001; Friedman and Voeltz, 2011; Hu et al., 2011; Westrate et al., 2015). Early descriptions from thin-section electron micrographs described the ER as an interconnected network of ribosome-studded “rough” membrane sheets and “smooth” tubules that both enclose a lumen of ∼60-nm diameter (Palade and Porter, 1954; Palade, 1955; Fawcett, 1981). More recently, advanced 3D EM approaches revealed ER membrane morphologies that diverge from these textbook descriptions. These ER morphologies include fenestrated sheets (Puhka et al., 2012), helicoidal membranes (Terasaki et al., 2013), “thin” cortical sheets (∼25 nm in thickness; Orci et al., 2009), and tubules that widen and narrow (diameters ranging from 25 to 90 nm; Terasaki, 2018). Light microscopy approaches further revealed that ER tubules are dynamic and constantly reorganize to facilitate the various functions of the ER (Dabora and Sheetz, 1988; Baumann and Walz, 2001; Hein et al., 2008; Westrate et al., 2015; Phillips and Voeltz, 2016; Guo, 2018; Holcman et al., 2018). Recent work from Nixon-Abell et al. (2016) used structured illumination microscopy (∼100-nm resolution) to identify “ER matrices,” dense and highly dynamic ER tubule networks that appear to be sheets by conventional microscopy. Collectively, these findings challenge the dogma that the peripheral ER consists of two distinct morphologies: flat sheets and curved tubules (Baumann and Walz, 2001; Shibata et al., 2006).

The high membrane curvature found in both ER tubules and rims of sheets requires the ER-specific wedge-shaped Reticulon and DP1/Yop1 family of membrane inserted proteins, which are excluded from flat membrane sheet regions (Voeltz et al., 2006; Shibata et al., 2008). The integral membrane protein Climp63 maintains the luminal space of ER sheets by forming bridges between parallel membrane sheets through its coiled-coil domain (Shibata et al., 2010). Although Climp63 overexpression induces ER sheets, the formation of ER sheets does not depend on Climp63. Instead, it is proposed that the abundance of Reticulon and DP1/Yop1 proteins relative to the amount of bilayer lipids determines the ratio of ER sheets to tubules (Shibata et al., 2010).

The microtubule cytoskeleton also functions to support the architecture of the ER (Waterman-Storer and Salmon, 1998; Grigoriev et al., 2008; Woźniak et al., 2009; Friedman et al., 2010). The depolymerization of microtubules causes ER tubules to coalesce into membrane structures that appear to be sheets by conventional light microscopy (Terasaki et al., 1986; Lu et al., 2009). Thus, although in vitro reticulons are sufficient to generate membrane tubules from proteoliposomes (Hu et al., 2008), microtubules are additionally required in vivo (Terasaki et al., 1986; Lu et al., 2009).

We used stimulated emission depletion (STED) microscopy (Hell and Wichmann, 1994) to survey the nanoscale morphology and dynamics of the ER at ∼50-nm resolution. We provide precise measurements of ER tubules from living cells. We characterize an understudied yet prominent feature of ER membranes—dynamic, nanoscale-sized holes in ER sheets that we call “nanoholes.” We demonstrate the effect of reticulons, Climp63, and the microtubule cytoskeleton on ER membrane structures that we conclude exist within a continuum between flat sheets and curved tubules.

## Results and discussion

**Figure 1.**
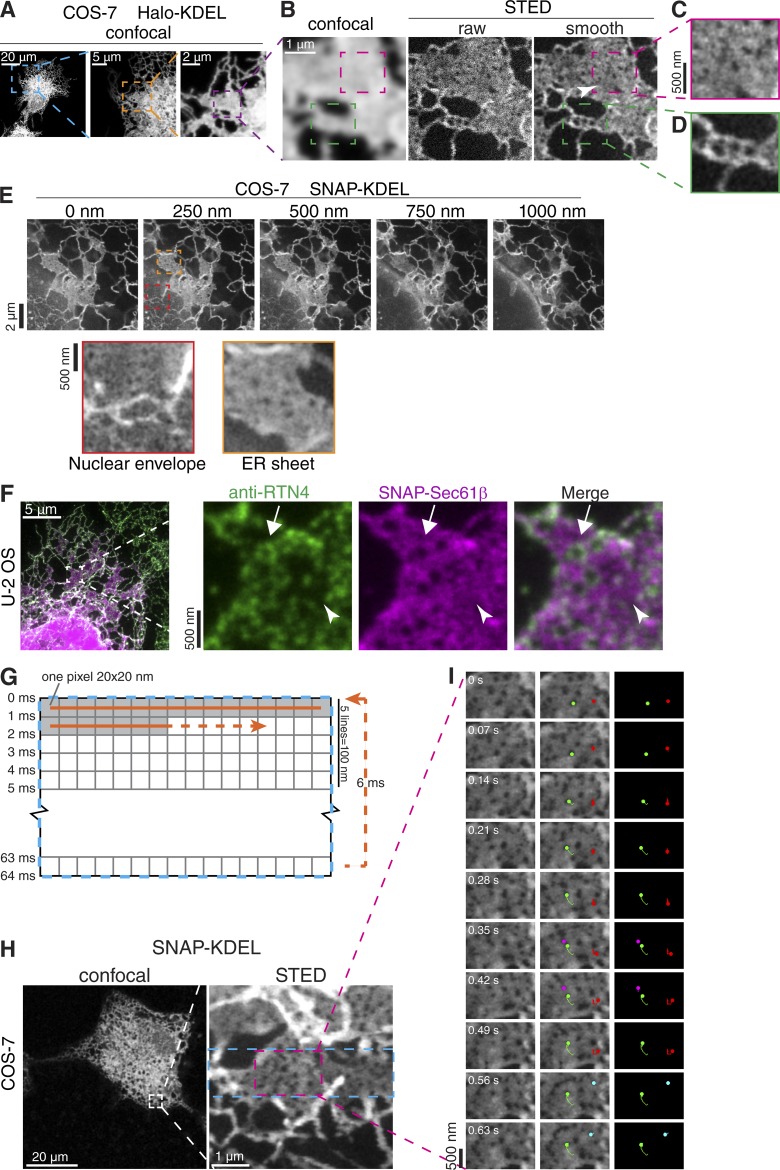
**Dynamic nanoscale-sized holes in ER sheets. (A–D)** Overview and magnified confocal and STED images of COS-7 cell expressing Halo-KDEL. White arrowhead in right panel of B shows uniform sheet region near holes (magenta box; magnified in C). Green boxed regions show clustered tubules. Boxed regions in subsequent magnified live images may be slightly shifted because of ER movements. **(E)** STED images of COS-7 cells expressing SNAP-KDEL acquired every 250 nm along the z axis near the NE. Magnified STED images show nanoscale-sized holes in the NE (red box) and ER sheet adjacent to the NE (orange box). **(F)** STED image of fixed U2OS cells expressing SNAP-Sec61Bβ (magenta) and immunolabeled for endogenous Rtn4B/D (green). Arrow in magnified image points to a nanohole that contains Rtn4, and arrowhead points to a uniform sheet region devoid of Rtn4. **(G)** STED image acquisition schematic. **(H)** Confocal image and magnified STED image of COS-7 cell expressing SNAP-KDEL. Blue rectangular region in magnified image outlines the 4.8 × 1.2-µm region imaged live with smaller magenta boxed region of the video shown in H. **(I)** Select STED images from time-lapse series paired with images pseudocolored to highlight manually tracked nanoholes during time course of video. *n* = 24 holes of *n* = 1 cell. Time shown in seconds.

We imaged the periphery of live COS-7 cells expressing the genetically encoded fusion protein Halo-KDEL or SNAP-KDEL (Keppler et al., 2003; Los et al., 2008), which exclusively localizes to the ER lumen and can be labeled with organic dyes compatible with STED imaging (Fig. 1 A; Bottanelli et al., 2016). Acquiring STED and confocal images of the same region within the cell periphery revealed ER structures that were not detected by conventional microscopy approaches (Fig. 1 B). In regions that appeared to be ER sheets by confocal microscopy, STED microscopy revealed sheets containing distinct holes directly adjacent to uniform regions within the same sheet (Fig. 1 B, right, magenta box; and Fig. 1 C). In most cases, sheets containing holes were distinct from clusters of tubules, which also appeared as sheets by conventional microscopy (Fig. 1 B, right, green box; and Fig. 1 D). Holes in ER sheets were observed with luminal as well as membrane ER markers (Fig. 1, A–E; and Fig. S1 A), in 3D image stacks of centrally located ER sheets—which are likely ribosome studded based on their proximity to the NE (Fig. 1 E)—and in U2OS cells (Fig. 1 F). Holes in the NE where nuclear pore complexes (NPCs) reside, as indicated by endogenously tagged nuclear pore protein Nup160-Halo, were similar in diameter to holes in ER sheets in both live and fixed cells (Fig. S1, B–E; and Fig. 1 E). Holes in ER sheets resemble fenestrated sheets first described by Palade (1955) over 60 yr ago and have since detected by high-resolution EM of tissue culture cells (Puhka et al., 2012) and budding yeast (West et al., 2011). We decided to focus on these nanostructures because despite their prominence, they are poorly characterized and have never been described in living cells. Because of their subdiffraction-limited size, we termed holes in ER sheets nanoholes.

To determine whether curvature-stabilizing reticulon proteins localize to curved membrane edges of nanoholes in ER sheets, we used two-color STED imaging of immunolabeled endogenous Rtn4A, Rtn4B, and Rtn4D isoforms (Fig. 1 F; henceforth Rtn4) in U2OS cells. Rtn4 localizes to a subset of nanoholes (Fig. 1 F, arrow) that juxtapose uniform sheet areas completely devoid of Rtn4 (Fig. 1 F, arrowhead). We endogenously tagged one locus of Rtn4 isoforms with a SNAP-tag in U2OS cells (Rtn4-SNAP^EN^; Fig. S1 F), but the fluorescence signal in STED microscopy was too low for robust analysis. Using confocal imaging instead, we confirmed Rtn4-SNAP^EN^ localization to a subset of nanoholes in otherwise flat ER sheets that were identified by STED imaging of the luminal marker Halo-KDEL (Fig. S1, G and H). Rtn4-SNAP^EN^ localization to nanoholes in flat sheets (Fig. S1 G) was distinct from membrane structures resembling clustered tubules that contain Rtn4-SNAP throughout (Fig. S1 H, arrow). We conclude that Rtn4 associates with nanoholes in otherwise uniform ER sheets.

To determine whether nanoholes are stable or dynamic features of ER sheets, we took advantage of the rapid scan speed of the STED microscope’s resonant scanner in bidirectional scan mode (16,000 scanned lines per second) to capture 4.8 µm × 100 nm regions in 5 ms (Fig. 1, G–I; and Video 1). By combining the high spatial resolution provided by STED with excellent local temporal resolution, this approach was comparable with imaging of similar regions in the ER by structured illumination (Fig. S1 I and Video 2) but provided the superior spatial resolution needed to clearly detect and track nanoholes (Fig. 1, H and I; and Video 1). This method shows that nanoholes can be extremely mobile in the plane of ER sheets (Fig. 1 I). Tracking individual holes (*n* = 24 holes of *n* = 1 cell), we found that approximately half of nanoholes persisted during the time course of the video. Surprisingly, the remaining half of tracked nanoholes appeared, disappeared, or both during the time course of the video (Fig. 1 I and Video 1). These data suggest that the majority of nanoholes are transient or change between diameters above and below our resolving power on subsecond time scales.

**Figure 2.**
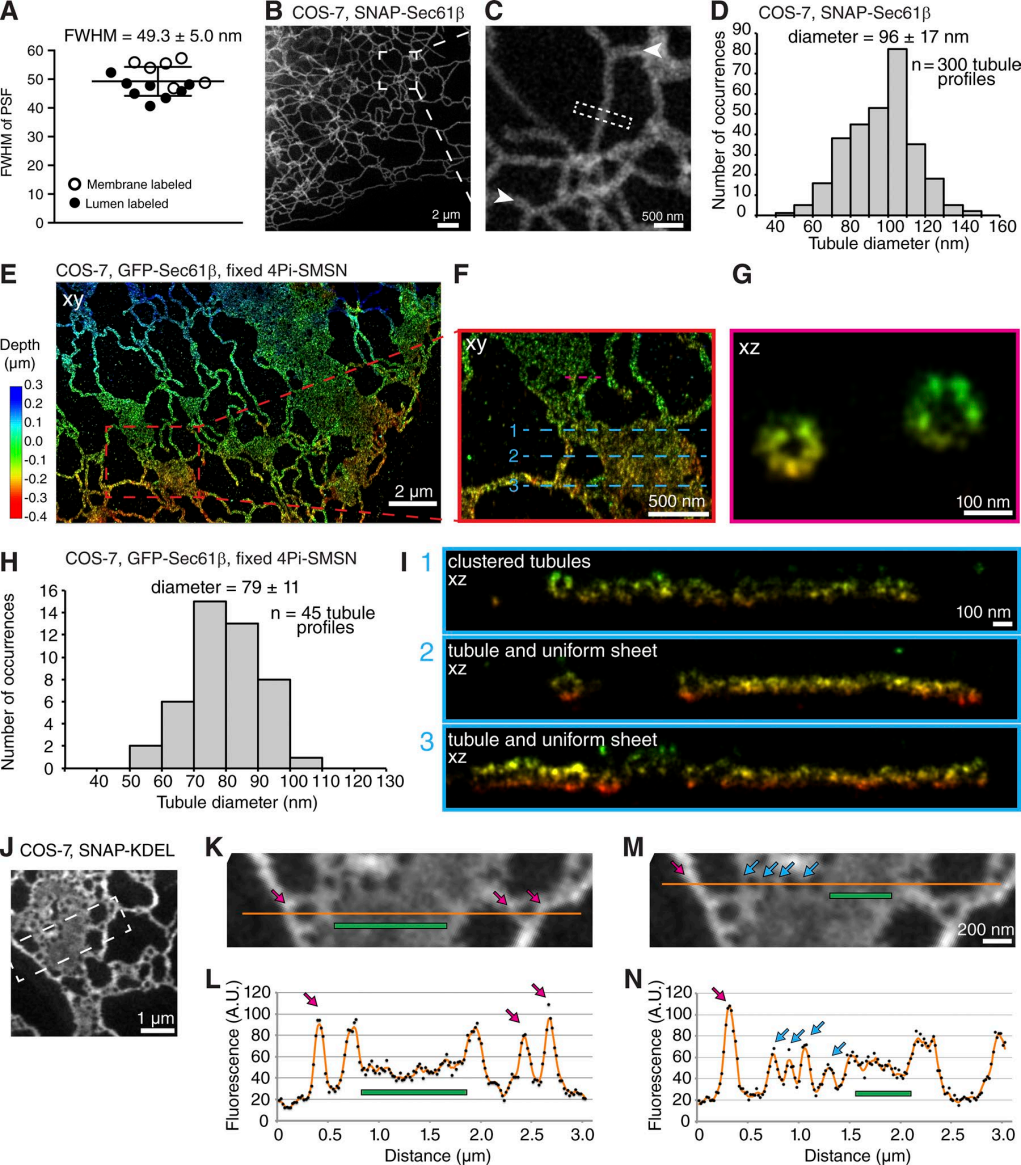
**Analysis of ER tubules, sheets, and nanoholes with superresolution microscopy. (A)** Plot of calculated PSF FWHM of STED microscope. Measurements taken from two cells per day; *n* = 4 imaging days; each point is from *n* = 50 profiles. FWHM, mean ± SD. **(B and C)** Live STED images of ER tubules labeled with SNAP-Sec61β. Arrowheads mark ER lumen. Dashed rectangle represents region used to generate fluorescence intensity line profiles to calculate tubule diameters in D. **(D)** Histogram of ER tubule diameters in living cells. *n* = 300 tubule profiles; *n* = 3 imaging days. 50 SNAP-Sec61β-labeled tubule profile measurements taken from each cell; two cells per day; diameter = mean ± SD. **(E–G)** Overview and cross sections of ER network in fixed COS-7 cell expressing GFP-Sec61β imaged with 4Pi-SMSN. Regions marked by dashed lines in xy view in F are shown as xz views of 20-nm-thick y slices shown in G and I. **(H)** Histogram of ER tubule diameters labeled with SNAP-Sec61β in fixed cell. *n* = 45 tubule profiles. Mean ± SD is shown. **(I)** xz view of 20-nm thick y slices of regions marked by blue dashed line in F. **(J–N)** Overview and magnified STED images of ER network. Orange line in K and M represents 5-pixel-wide fluorescence intensity line profile shown in L and N. Magenta arrows mark ER tubules, green bars mark uniform ER sheets, and cyan arrows mark ER regions flanking nanoholes. Line profiles in L and N are from raw image (black dots), and image was smoothed with a uniform 3 × 3 filter (orange line). Traces are from one cell.

To accurately characterize nanohole architecture in the context of other ER structures, we measured the resolution provided by STED microscopy. Rather than relying on measuring bead samples that are not directly comparable with our live-cell imaging experiments, we used a recently developed method that determines the resolution of STED images of living cells from the image itself (Barentine et al., 2018). Our measurements determined a STED xy resolution of 49.3 ± 5.0 nm (mean ± SD; Fig. 2 A; full width at half maximum [FWHM] of the point-spread function [PSF]), compared with ∼250-nm confocal resolution. This resolution facilitates the visualization of nanoholes in ER sheets (Fig. 1 B, right, magenta box; and Fig. 1 C) as well as clustered ER tubular structures (Fig. 1 B, right, green box; and Fig. 1 D) also observed by structured illumination microscopy (Nixon-Abell et al., 2016). Accounting for both the expected labeling of the structure, and the PSF of the microscope, we accurately determined the diameter of ER tubules in living cells (Fig. 2, B–D; and Fig. S2, A and B; Barentine et al., 2018). We found that membrane-labeled ER tubules in live COS-7 cells are 96 ± 17 nm in diameter (mean ± SD; *n* = 300 tubules) with values as small as 48 nm and as large as 144 nm (Fig. 2 D). Using the same analysis on ER tubule data with luminal labeling yielded consistent diameters (Fig. S2, A and B). This range in values is in agreement with measurements of membrane-labeled tubules in fixed cells imaged at 20-nm resolution in x, y, and z dimensions with 4Pi single-molecule switching nanoscopy (4Pi-SMSN; Fig. 2, E–I; 79 ± 11 nm; mean ± SD; as small as 54 nm and as large as 101 nm; *n* = 45 tubules in Fig. 2 H; Huang et al., 2016). Accounting for 20% shrinkage from solvent dehydration used in EM sample preparation, our STED and 4Pi-SMSN measurements are close to the ER tubule dimensions in EM samples (Terasaki [2018] reported ER tubules in the intercalated ducts of mouse salivary glands frequently 60 nm diameter but ranged from ∼25 to ∼90 nm). Additionally, examination of xz views of ER sheets and tubules in 4Pi-SMSN images corroborated that ER tubules can have different diameters (Fig. 2 G) as well as the presence of ER regions made up of clustered tubules (Fig. 2 I, top, xz view).

We noticed that compared with the diameter of tubules, the luminal width between membrane sheets imaged by 4Pi-SMSN appeared markedly narrower (Fig. 2 I, xz views). This observation is in agreement with the difference in signal intensity in sheets versus tubules in STED images of SNAP-KDEL: the signal intensity of SNAP-KDEL is roughly proportional to the thickness of the luminal volume because the z resolution of the STED microscope over which the signal is accumulated in the depth direction is much larger than the thickness of a single ER tubule or sheet (∼600 nm z resolution versus 50–140 nm ER lumen thickness in Fig. 2 D; Eggeling et al., 2013). Line profiles drawn across images of ER sheets and tubules show clear differences in the SNAP-KDEL signal intensity between areas identifiable as uniform sheets and tubules (Fig. 2, J–L). The observed lower signal in ER sheets versus tubules (Fig. 2, K and L) suggests that the thickness of ER sheets in living cells is ∼30–50 nm (Fig. S2, C and D), which is consistent with narrow ER sheet thickness in samples imaged by 4Pi-SMSN (Fig. 2 I) and previous EM studies on fixed mammalian samples (55 ± 9.3 nm, Shibata et al., 2010; 48 ± 8 nm helicoid sheets, Terasaki et al., 2013; 24 ± 0.4 nm thin cortical ER, Orci et al., 2009).

We used the signal intensity of SNAP-KDEL to confirm that nanoholes are within uniform sheets and not gaps between tubules. The intensity of regions between nanoholes is similar to the signal intensity of uniform sheets and usually lower than the signal intensity of tubules (Fig. 2, M and N). Additionally, the distance between nanoholes varies over a wide range and is usually larger than the diameter of a tubule (Fig. S2, E–I). The minimum intensity within nanoholes is well above background level compared with gaps between tubules, indicating the small size of nanoholes (Fig. S2, F–I). Together, these data, combined with high spatial resolution that resolves gaps larger than a few tens of nanometers, and temporal resolution that shows persistent nanoholes, provide further evidence that we can detect nanoholes in ER sheets in living cells.

**Figure 3.**
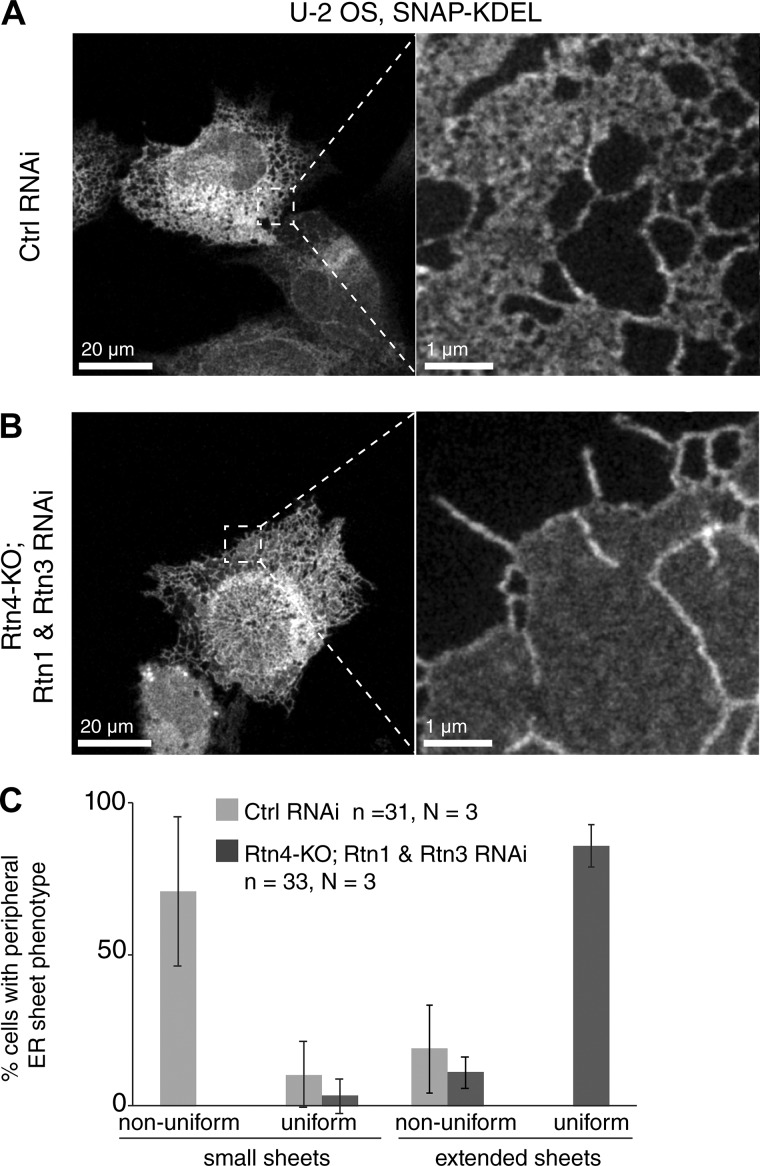
**Uniform sheets devoid of nanoholes form in the absence of reticulons. (A and B)** Overview confocal and magnified STED images of SNAP-KDEL–labeled ER network in control and CRISPR-Cas9–KO Rtn4 U2OS cells under indicated RNAi conditions. **(C)** Plot represents percentage of cells with indicated ER sheet phenotypes (nonuniform, detectable nanoholes; uniform, no detectable nanoholes) for indicated conditions. Error bars show standard deviation. No statistical significance test was performed.

To test whether reticulons have a role in generating and/or maintaining nanoholes in ER sheets, we used CRISPR-Cas9 technology to generate Rtn4 knockout (KO) human U2OS cells (Fig. S2 J) and codepleted the redundant Rtn1 and Rtn3 using siRNA (Figs. 3 and S2 K). Cells lacking reticulons formed large sheets that extend to the edge of the cell and a few remaining tubules (Figs. 3 B and S2 K), similar to what has been previously reported (Anderson and Hetzer, 2008; West et al., 2011). ER sheets generated in the absence of reticulons were uniform and completely devoid of nanoholes (Fig. 3, B and C). The stark contrast between Fig. 3 B and images of nonuniform sheets with nanoholes (Figs. 3 A and 1, B–F and H) highlights the ability of the microscope and SNAP-KDEL labeling to reliably discern uniform sheets from those with nanoholes. These data, together with the localization of Rtn4 to curved edges of nanoholes (Fig. 1 F), demonstrate that reticulon family members are essential for the formation and/or stability of curved edges surrounded by membrane sheets of the ER to generate nanoholes.

**Figure 4.**
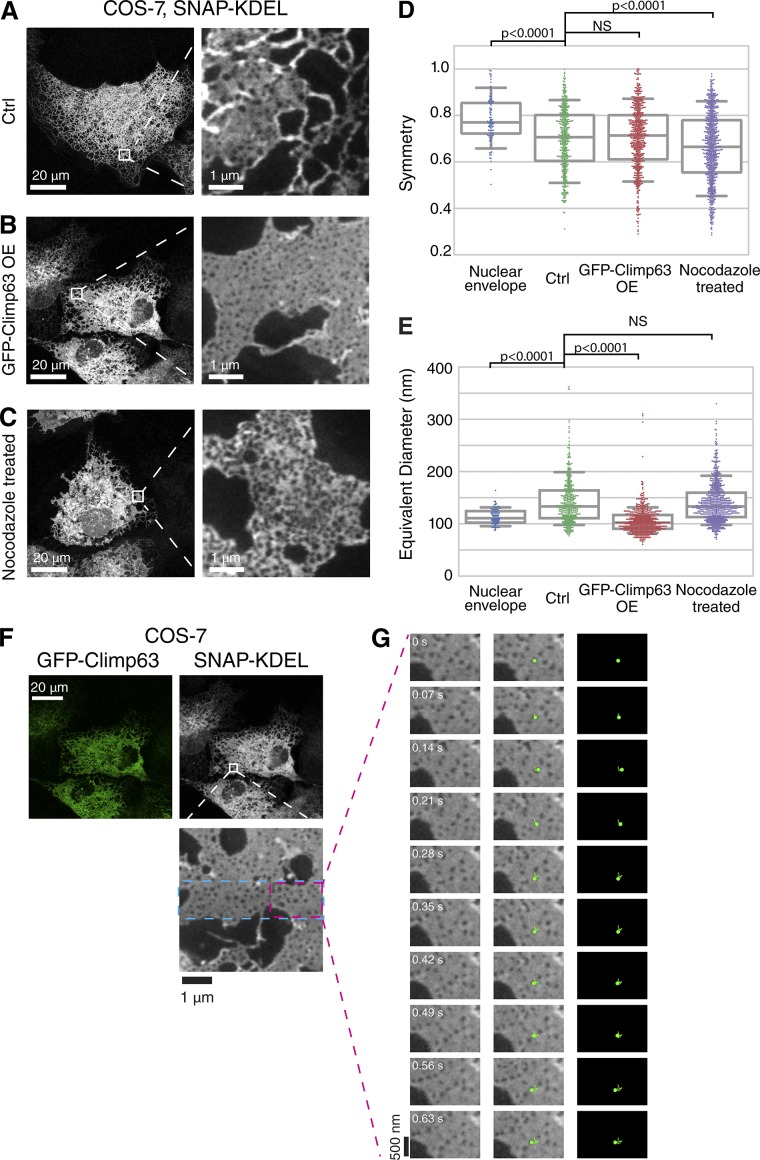
**Analysis of the role of Climp63 and microtubules on the nanoscale architecture of ER membranes. (A–C)** Confocal images of SNAP-KDEL in living COS-7 cells (left) and STED image of magnified boxed region (right) in indicated conditions. **(D and E)** Plot of hole symmetry (unitless) and hole-equivalent diameter in indicated conditions. Median and interquartile range are shown with whiskers drawn down to the 10th percentile and up to the 90th percentile. Median and interquartile range for hole symmetry: NE, 0.77, 0.13; Unperturbed, 0.71, 0.21; GFP-Climp63 overexpressing (OE), 0.71, 0.19; and nocodazole treatment, 0.66, 0.23. Median and interquartile range for diameter in nm: NE, 111, 22; Unperturbed, 133, 53; GFP-Climp63 overexpression, 102, 26; and nocodazole treatment, 133, 47. Measurements for each plot were taken from NE, *n* = 97 holes, *n* = 3 cells; Unperturbed, *n* = 424 holes, *n* = 2 cells; GFP-Climp63, *n* = 601 holes, *n* = 3 cells; and nocodazole treatment, *n* = 713 holes, *n* = 4 cells. Kruskal–Wallis test with a post hoc Dunn’s test for multiple comparisons determined significance. **(F)** Overview confocal images and magnified STED image of ER network in a living COS-7 cell expressing GFP-Climp63 (green) and SNAP-KDEL (grayscale). Dashed blue line in magnified STED image outlines the 4.8 × 1.2-µm region imaged over time. The region outlined by the dashed magenta boxed is shown in G. **(G)** Select STED images paired with pseudocolored image and image overlay (third column) to highlight a manually tracked nanohole that persists over time. Time shown in seconds.

To gain a more comprehensive understanding of how other ER shaping proteins and the microtubule cytoskeleton affect the nanoscale architecture of the ER, we developed a semiautomated algorithm using watershed transformation that detects hole borders to measure the size and shape of holes within the ER (Fig. 4, A–E; and Fig. S3, A–F). As a reference, we applied the algorithm to analyze holes in the NE that contain NPCs (Fig. S1, B and C). The shape and size of holes in ER sheets varied greatly compared with the tight distribution of NE holes where NPCs are inserted (Fig. 4, D and E; and Fig. S3, A–D). This difference suggests that nanoholes in ER membrane sheets are regulated by mechanisms that are distinct from those responsible for stabilizing NPCs in the NE.

Next, we tested how changing the abundance of sheets relative to tubules affects the nanoscale architecture of the ER, with a focus on the distribution of the sizes and shapes of holes within the ER. Holes were manually identified, only excluding large holes several hundred nanometers in diameter bounded by tubules and those that segmented poorly (Fig. S3, E and F). We increased sheet abundance by overexpressing the intraluminal spacer Climp63 (Shibata et al., 2010) or treating cells with the microtubule-depolymerizing drug nocodazole. In control COS-7 cells, flat ER sheets that juxtaposed nanoholes had dim SNAP-KDEL intensity relative to the increased brightness at thicker sheet edges and tubules (Fig. 4 A, right) as observed before (Fig. 2, K–N). Proliferated sheets induced by Climp63 overexpression were rife with nanoholes and featured remarkably uniform SNAP-KDEL signal levels in areas between nanoholes and at the sheet edge, indicating even ER thickness (Fig. 4 B; compare with Figs. 4 A or 2 J). Our analysis of hole diameters showed that holes in Climp63-overexpressing cells were close in size to holes in the NE (median values 102 and 111 nm, respectively, vs. 133 nm in unperturbed cells; Fig. 4, A, B, and E). Endogenously tagged Rtn4-SNAP^EN^ localized to nanoholes in Climp63-induced sheets and was absent from flat membrane regions (Fig. S3, G–I).

In contrast, STED images of nocodazole-treated cells revealed ER structures with varying SNAP-KDEL intensity throughout (Fig. 4 C). These structures included regions with bright, uniform KDEL intensity (indicating uniformly thick sheets), dimmer regions with nanoholes (indicating sheets with nanoholes), and regions with bright SNAP-KDEL intensity that contained holes directly adjacent to each other (indicating a packed tubular network). The broader distribution of hole symmetry (Fig. 4 D) and the localization of Rtn4 throughout the network (Fig. S3, J–L) further reflect the change in ER morphology in the absence of microtubules. The changes in ER morphology revealed by STED are in contrast with the long-assumed view based on conventional light microscopy approaches that depolymerization of microtubules causes the tubular ER to coalesce into sheets (Terasaki et al., 1986; Lu et al., 2009). We conclude that Climp63 and microtubules modulate the distribution of ER membrane structures within a continuum that includes flat sheets with nanoholes and packed ER tubular networks and that at the extremes forms uniform sheets and tubules.

With regard to dynamics, we reasoned that increasing the abundance of Climp63, which has been reported to multimerize into clusters with low mobility (Klopfenstein et al., 2001), may corral nanoholes in ER sheets and limit their ability to travel in the plane of the sheet. We were able to manually track a greater number of nanoholes in Climp63-induced sheets than in untreated cells (Fig. 4, F and G; compared with Fig. 1, H and I), and a greater percentage of nanoholes persisted over the imaging time course (80%; *n* = 59 holes of *n* = 1 cell; Fig. 4 G and Video 3). These data indicate that the dynamics of nanoholes in ER sheets are strongly influenced by Climp63. The increased persistence of nanoholes in ER sheets induced by Climp63 overexpression provides further evidence that nanoholes in ER sheets can be distinguished from highly dynamic tubules that form matrices in the cell periphery (Nixon-Abell et al., 2016).

We have defined the nanoscale architecture of the ER using live-cell STED to establish a baseline for the morphology and dimensions of ER membranes in living cells. The xy resolution provided by STED microscopy (∼50 nm) reveals ER membrane structures that are otherwise not visible by confocal microscopy. Our images show that the peripheral ER is not limited to uniform sheets and tubular networks but contains a continuum of diverse membrane structures including dynamic nanoholes in ER sheets that coexist with uniform regions. Nanoholes are equivalent to fenestrations in fenestrated ER sheets that were first introduced by Palade (1955) over 60 yr ago. Our STED microscopy approach unequivocally demonstrates that nanoholes exist in ER sheets in living cells. Our observation agrees with more recent research showing the presence of fenestrated ER sheets using serial block face scanning EM (Puhka et al., 2012).

Our images reveal the complete loss of nanoholes in cells deleted of Rtn4 then codepleted of Rtn1 and Rtn3. These data, combined with the fact that Rtn4 localizes to the curved edges of nanoholes in ER sheets, indicate that reticulons are essential to generate and/or maintain nanoholes in membrane sheets of the ER. Given that Rtn4 is a curvature stabilizing protein (Voeltz et al., 2006), its presence in nanoholes in the middle of a sheet may contribute to determining and stabilizing sheet thickness.

**Figure 5.**
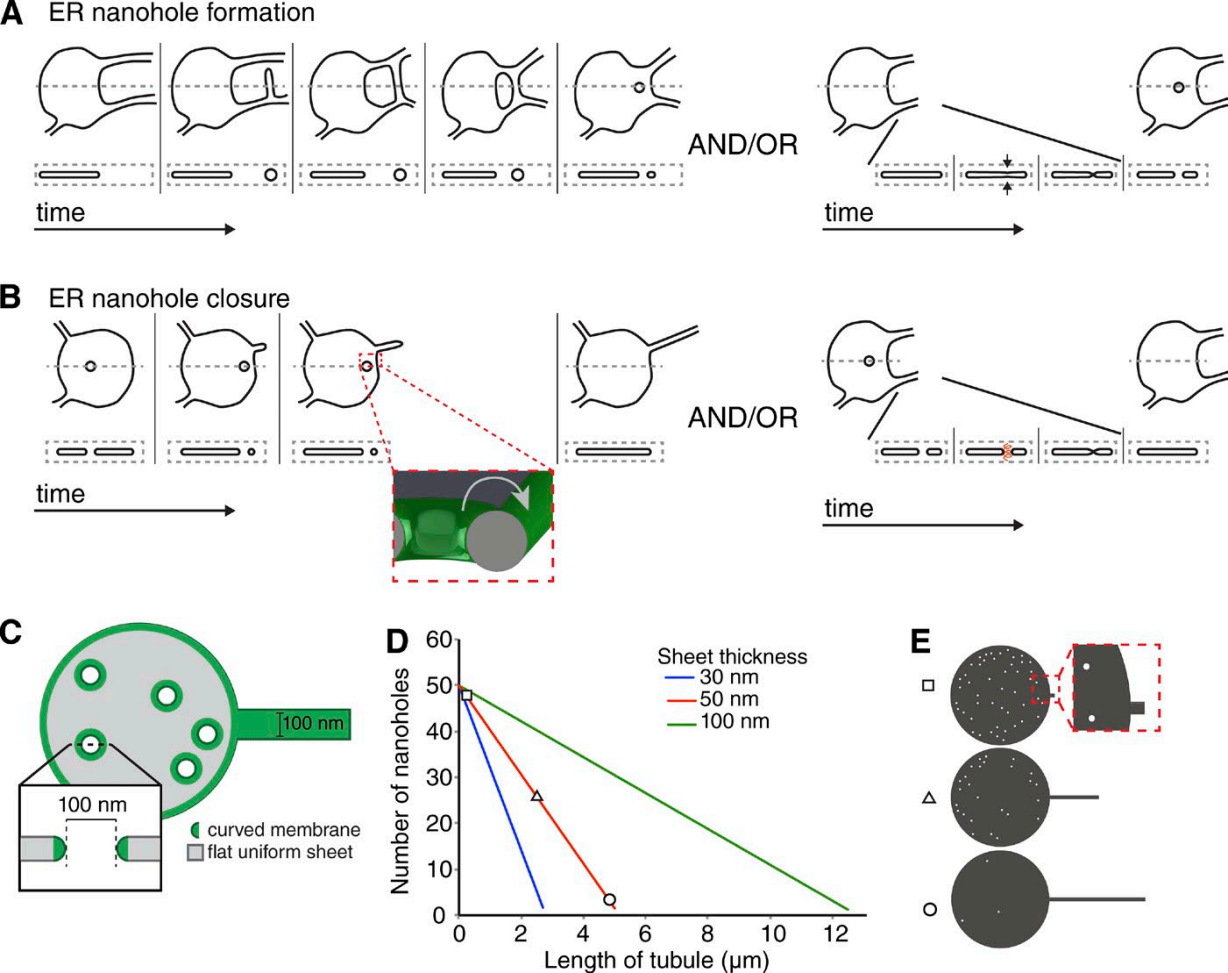
**Model for function and formation/closure of nanoholes. (A)** Schematic of possible mechanisms for nanohole formation. Top view of ER sheet with a dashed gray line indicating the region shown as a side profile below. Nanohole formation by loops that shrink (left) and/or fusion of membrane sheets across the ER lumen, generated or stabilized by reticulons. **(B)** Schematic of possible mechanisms for nanohole closure. Diffusion of curvature-stabilizing proteins to sheet edge and subsequent tubule extension (left). Region marked by dashed red box is displayed as a tilted 3D structure to show that when nanoholes approach the edges of a sheet, the curved edge of a nanohole will be directly adjacent to the curved sheet edge. Potential mechanism of nanohole closure by fission machinery (right). **(C)** Schematic of geometries used to model the ER for nanohole function in tubule extension. **(D)** Plot of curved fraction of surface area as number of holes and tubule length are varied with the total surface area held constant. **(E)** Three points on the red isocline in D, coordinates marked with square, triangle, and circle, are drawn to scale with the appropriate number of holes and length of tubule.

The fact that nanoholes have sheet-like as well as tubule-like morphological features allows for a diverse range of mechanisms to explain how they are created and resolved. In addition to machinery that might facilitate membrane fusion or fission similar to those postulated for NPC insertion and hole closure in the NE (Rothballer and Kutay, 2013; Otsuka et al., 2016; Vietri et al., 2016), processes that enable ER tubule fusion and fission may control nanohole creation or removal within sheets (see model in Fig. 5, A and B; Hu et al., 2009; Orso et al., 2009; Shibata et al., 2009). Future work is required to unravel the mechanism of nanohole formation and closure and determine the role of reticulons in this process.

We show that nanoholes in ER sheets are comparable in their dimensions to holes in NE occupied by NPCs, but unlike NPCs, they can take on a large range of shapes and diameters. Recent work showed that NPCs assemble in preexisting holes in ER-derived nuclear membranes during NE reformation in mitosis (Otsuka et al., 2018). It is attractive to consider that nanoholes in ER sheets provide holes for NPC assembly upon mitotic exit. In support of this idea, reticulons have been proposed to stabilize holes in the NE before NPC assembly (Anderson and Hetzer, 2008; Dawson et al., 2009), and holes in ER sheets have been observed in fixed mitotic cells (Puhka et al., 2007, 2012).

The fact that reticulons are able to form ER tubules as well as nanoholes leads us to speculate that nanoholes in ER sheets could act as a readily available reservoir for curvature-stabilizing proteins, such as Rtn4, that facilitate tubule formation and thereby support morphological changes to the ER more quickly than through protein or lipid synthesis or interorganelle transfer. To investigate how the amount of curved membrane changes as a function of its morphology, we developed a simple geometric model of the ER consisting of a single disk-shaped sheet with an adjustable number of nanoholes (100-nm inner diameter) and a 100-nm-diameter ER tubule of variable length extending from it (Fig. 5 C). We assumed 30-, 50-, and 100-nm-thick ER sheets. We exclude lipid synthesis or interorganelle transfer from our model by assuming that the total surface area of the modeled ER is constant and any change in surface area resulting from tubule or nanohole addition is compensated by an adjustment of the sheet disk diameter, which is initially 5 µm. Using this geometric model with a 50-nm-thick sheet, we determined that 10 nanoholes store the same curved membrane area as ∼1 µm of ER tubule (Fig. 5, C–E). These calculations show that a modest number of nanoholes contain sufficient curvature to support tubule extension or retraction without changes in the total number of curvature-stabilizing proteins or lipids.

Thus, in addition to the abundance of curvature stabilizing proteins and bilayer lipids in dictating ER morphology (Shibata et al., 2010), nanoholes may provide a way to alter local ER morphology and dynamics on rapid time scales. The high mobility of nanoholes allows for their rapid diffusion to the sheet edge so that reticulons stored in nanoholes could easily assemble onto growing ER tubules or morph into tubular matrices. Altering local ER morphology by adding or removing nanoholes may be important to ER functions that are specific to the cell periphery, especially at locations far away from the site of transcription or translation, such as at remote neuronal processes, and offers a possible mechanism to quickly react to cellular demands.

In conclusion, we demonstrate the existence of dynamic nanoscale-sized fenestrations in ER sheets for the first time in living cells. Our data show that nanoholes in sheets are distinct from uniform sheets and ER matrices (Nixon-Abell et al., 2016). We suggest that nanoholes represent an element of a local continuum of membrane structures that make up subdomains of the ER, providing an explanation for the ER membrane morphologies described by others in fixed cells (Palade, 1955; Puhka et al., 2007, 2012; West et al., 2011) and in live cells (Nixon-Abell et al., 2016) that diverge from textbook definitions of ER sheets and tubules. Additionally, we find that ER sheets are present at the cell periphery as has been reported by others using conventional and EM approaches (Shibata et al., 2009, 2010; Puhka et al., 2012; Terasaki et al., 2013). ER morphology is linked to human disease (Westrate et al., 2015), and thus the prominence of nanoholes as a membrane feature of the ER in living cells provides a more comprehensive view of ER structure that may contribute to our future understanding of the relationship between ER structure and disease.

## Materials and methods

### Cell culture

COS-7 (CRL-1651; ATCC) cells were grown in phenol red–free DMEM (Gibco) + 10% FBS (Gibco) in a standard mammalian cell incubator. U2OS (HTB-96; ATCC) cells were grown in phenol red–free McCoy’s 5A medium (GE Healthcare) + 10% FBS. U2OS cells stably expressing GFP-Sec61β were provided by T. Rapoport (Harvard University, Boston, MA). All cells were cultured using 0.05% trypsin (Gibco) to split and subculture. DNA plasmids were transfected using Super Electroporator NEPA21 Type II (Nepa Gene). Electroporation was performed on 10^6^ cells that were suspended in OptiMEM (Gibco) and 1–10 µg DNA, depending on the desired expression level, in electroporation cuvettes that had a 2-mm gap (12358-346; Bulldog Bio). Cells were electroporated using the following program: 125-V poring pulse, 3-ms pulse length, 50-ms pulse interval, two pulses, with decay rate of 10% and + polarity, followed by a 25-V transfer pulse, 50-ms pulse length, 50-ms pulse interval, five pulses, with a decay rate of 40% and ± polarity. Cells were imaged 12–48 h after electroporation.

In microtubule destabilization experiments, cells were treated with 33 µM nocodazole for 30 min at 37°C in a mammalian cell incubator. Samples were imaged for up to 30 min in the presence of nocodazole so no cells were exposed to 33 µM nocodazole for >1 h.

### Plasmids

Luminal ER labeling plasmids were made from pDsRed2-ER (Takara Bio Inc.), which contains a signal sequence preceding the DsRed2 gene fused to the sequence encoding the tetrapeptide KDEL. The Halo-KDEL plasmid was made by removing the sequence for DsRed2 using AgeI and HindIII restriction endonucleases (New England Biolabs). The HaloTag7 (Halo) sequence was amplified using the primers 5′-ATACCGGTCGATGGCAGAAATCGGTACTGGCTTTC-3′ and 5′-CTGAAGCTTTTACAGCTCGTCCTTCTTGCCGGAAATCTCGAGCGTC-3′, digesting both amplified Halo and pDsRed2-ER using AgeI and HindIII, and ligating Halo into the larger pDsRed2-ER backbone fragment. The SNAP-KDEL plasmid was made similarly using the primers 5′-ATACCGGTCGATGGACAAAGACTGCGAAATGAAGCG-3′ and 5′-CTGAAGCTTTTACAGCTCGTCCTTCTTACCCAGCCCAGGCTTGC-3′ to amplify SNAP-tag (SNAP). SNAP-Sec61β and Halo-Sec61β were made as reported by Bottanelli et al. (2016). GFP-Sec61β was a gift from M. Davidson (deceased, formerly Florida State University, Tallahassee, FL; Addgene plasmid 54249; mEmerald-Sec61-C-18). GFP-Climp63 (AcGFP1-MsClimp63) was provided by T. Rapoport.

### Reagents

For information on reagents, see Table S1.

### CRISPR/Cas9 genome editing

All guide RNA (gRNA) sequences were designed using the online CRISPR tool (http://crispr.mit.edu), which reported no off-target matches (protospacer-adjacent motif [PAM] sequence is underlined): RTN4^EN^-SNAP, 5′-AAACGCCCAAAATAATTAGTAGG-3′; Nup160^EN^-Halo, 5′-CACGGGATTTATTATATCGTCGG-3′; and RTN4 KO, 5′-CGTTCAAGTACCAGTTCGTGAGG-3′. For *RTN4^EN^-SNAP*, the gRNA (listed above) was chosen to target the RTN4 genomic locus (gene ID: 57142) directly after the stop codon. The gRNA sequence was synthesized as part of a 445-bp gBlock of DNA (Mali et al., 2013), which contains a U6 promoter, target sequence, gRNA scaffold, and termination sequence required for gRNA expression with the following sequence: 5′-TGTACAAAAAAGCAGGCTTTAAAGGAACCAATTCAGTCGACTGGATCCGGTACCAAGGTCGGGCAGGAAGAGGGCCTATTTCCCATGATTCCTTCATATTTGCATATACGATACAAGGCTGTTAGAGAGATAATTAGAATTAATTTGACTGTAAACACAAAGATATTAGTACAAAATACGTGACGTAGAAAGTAATAATTTCTTGGGTAGTTTGCAGTTTTAAAATTATGTTTTAAAATGGACTATCATATGCTTACCGTAACTTGAAAGTATTTCGATTTCTTGGCTTTATATATCTTGTGGAAAGGACGAAACACCGAACGCCCAAAATAATTAGTGTTTTAGAGCTAGAAATAGCAAGTTAAAATAAGGCTAGTCCGTTATCAACTTGAAAAAGTGGCACCGAGTCGGTGCTTTTTTTCTAGACCCAGCTTTCTTGTACAAAGTTGGCATTA-3′. This gBlock was amplified to increase DNA quantity with primers that anneal to the first and last 20 bp of the above sequence.

A homology-dependent repair (HDR) template was generated to add linker and SNAP sequences to the *RTN4* gene, fusing SNAP tag to the C terminus of all RTN4 isoforms (Fig. S1 F). The HDR template was engineered using pSNAP-N1 as the starting plasmid. The pSNAP-N1 was synthesized by amplifying SNAP with the forward primer 5′-CTACCGGTCGCCACCATGGACAAAGACTGCGAAATGAAGC-3′ and reverse primer 5′-TCGCGGCCGCTTTAACCCAGCCCAGGCTTGC-3′. The SNAP gene was then digested with AgeI and NotI (New England Biolabs) and subsequently ligated into peGFP-N1, which had the GFP gene removed when it was digested with the same restriction endonucleases. The left homology arm, containing ∼1 kb of genomic sequence upstream of the cut site and the linker 5′-GGTGGTTCTGGTGGTGGTTCTGGT-3′ (modified from http://parts.igem.org/wiki/index.php/Part:BBa_K243006), was amplified and inserted into NdeI and ApaI (New England Biolabs) linearized pSNAP-N1 using Gibson assembly. The right homology arm, containing ∼1 kb of sequence downstream of the genomic cut site, was inserted into BsaI (New England Biolabs) linearized plasmid from the previous step using Gibson assembly. The right homology arm was inserted into the plasmid after the Neo/Kan resistance cassette, allowing the selection of successful recombination using the drug G418/Geneticin (Gibco). The exonic region in the HDR template plasmid identical to the PAM site of the gRNA was changed to create a silent mutation, preventing repeated cutting by Cas9 after HDR.

pSpCas9 (PX165) was a gift from F. Zhang (Massachusetts Institute of Technology, Cambridge, MA; Addgene plasmid 48137; Ran et al., 2013). U2OS cells were simultaneously transfected using Lipofectamine 2000 (Invitrogen) with PX165, gRNA dsDNA gBlock, and the HDR template. G418 was added to the growth media 1 wk after transfection. After 2 wk of selection, cells with successful recombination were labeled with SNAP-cell 647-SiR (SiR-BG; S9102S; New England Biolabs) and isolated using FACS to obtain single clones. Clones were genotyped via immunoblot and PCR.

### NUP160^EN^-Halo

The NUP160 genomic locus (gene ID: 23279) was targeted directly after the stop codon. The gRNA sequence (listed above) was synthesized as two oligonucleotides with BbsI overhangs and an additional guanidine base 5′ to the protospacer sequence, and the oligonucleotides were phosphorylated with calf alkaline intestine phosphatase (M0290; New England BioLabs) and annealed by heating to 95°C and cooling to room temperature. The annealed oligonucleotides were cloned into pSPCas9(BB)-2A-Puro (PX459) v2.0 (a gift from F. Zhang; Addgene plasmid 62988; Ran et al., 2013) that had been digested with BbsI-HF (R3539; New England BioLabs). The HDR template was generated to add a five-glycine linker and HaloTag7 sequence to the 3′ end of the *NUP160* gene, fusing Halo to the C terminus of all NUP160 isoforms. The HDR template was engineered using a four-piece Gibson assembly: (1) the vector backbone was pEGFP-N1 (Takara Bio Inc.) amplified with the following oligonucleotides so that the CMV promoter, MCS, and GFP sequence had been removed: forward primer 5′-CCTCCCCCTGAACCTGAAAC-3′ and reverse primer 5′-GGCTATGAACTAATGACCCCGT-3′; (2) the left homology arm, a gBlock containing 800 bp upstream of the NUP160 stop codon with a silent mutation to the PAM sites of one candidate gRNA and a 5′-GGAGGCGGCGGCGGC-3′ linker and was flanked by 20-bp overhangs that overlapped with the pEGFP-N1 backbone and HaloTag7; (3) HaloTag7 was amplified using the following oligonucleotides (a silent mutation in aspartic acid was included in the forward primer to facilitate amplification of the gene): forward primer 5′-ATGGACCCGAAATCGGTACT-3′ and reverse primer 5′-GCCGGAAATCTCTAGCGTC-3′; and (4) the right homology arm, a gBlock containing 800 bp downstream of the NUP160 stop codon with two point mutations in the PAM sites of candidate gRNAs that was flanked by 20-bp overlap with HaloTag7 and the EGFP-N1 backbone.

The PX459 v2.0 vector containing the NUP160 guide and the HDR template were transfected into U2OS cells using Lipofectamine 2000 and treated with 3 µg/ml puromycin (Invitrogen) in DMEM low glucose with 10% FBS for 48 h. The cells were labeled with HaloTag TMR ligand (G8251; Promega) and sorted for the top 1% of fluorescent cells. These cells were plated at <100 cells/ml into 10-cm dishes and grown in antibiotic-free DMEM low glucose with 10% FBS for 2 wk. Clonal cell lines isolated as colonies were trypsinized, picked with 1/8-inch sterile cloning discs (Bel-Art), and grown to confluence in a T75 flask, after which the genomic DNA was harvested using QiaAmp Mini kit (Qiagen). Two primer sets were used to amplify regions containing the NUP160 genomic locus using the same forward primer 5′-AGCAGTTACACCTTACAGCTTG-3′ (anneals upstream of left homology region) and either reverse primer 1 5′-AGGACTTCCACATAATGGGG-3′ (anneals to HaloTag7) or reverse primer 2 5′-AACTCAAGAAGGGTCAAAAGGCT-3′ (anneals downstream of right homology region). Clonal cell lines that exhibited an integration of the HaloTag as shown by PCR using reverse primer 1 as well as lack of the WT allele as shown by PCR using reverse primer 2 were chosen for imaging. Protein lysates of the bulk and clonal populations were run on an 8% polyacrylamide gel and transferred to a nitrocellulose membrane for Western blot with rabbit anti-HaloTag (G9281; Promega) to confirm that the tagged protein is full length.

### RTN4 KO

The gRNA sequence (listed above) is located near the beginning of the reticulon homology domain common to all isoforms of RTN4 (gene ID: 57142) in the first exon of RTN4C. The guide was cloned into PX459 v2.0 as described for NUP160^EN^-Halo. This vector was transfected into U2OS cells using Lipofectamine 2000 and selected with 3 µg/ml puromycin (Invitrogen) for 48 h. The remaining cells were grown, and bulk population genomic DNA was isolated for genotyping by sequencing and screening for indels by Tracking of Indels by Decomposition (TIDE) deconvolution (Brinkman et al., 2014) using the primers listed below. Once indels were detected in the bulk population, the cells were plated at <100 cells/ml into 10-cm dishes and grown in antibiotic-free DMEM with 10% FBS for 2 wk. Colonies of clonal populations were trypsinized, picked with 1/8-inch sterile cloning discs (Bel-Art), and grown in 24-well plates until cells could be harvested for genomic DNA harvesting and PCR analysis using PCR with the forward primer 5′-TTCGTGGTCAAAAATAAAGGTGTT-3′ and reverse primer 5′-TCCTCATCAAACCTACCCATGTT-3′. The clonal cell line used in experiments had a 5-bp deletion in >80% of alleles and 0% WT alleles as determined by TIDE deconvolution of sequencing. Cell lines also showed no expression of RTN4 B or D by immunoblot (Fig. S2 J).

### RNAi

WT or Rtn4-KO U2OS cells were plated into 35-mm imaging dishes (250,000 cells/dish). After 20 h, the WT cells were transfected with 80 nM Ambion Silencer Select negative control 1 siRNA (4390843), and the Rtn4KO cells were treated with 40 nM RTN1 Ambion Silencer Select Pre-designed siRNA (s12378) and 40 nM RTN3 Ambion Silencer Select Pre-designed siRNA (s20162) according to the DharmaFECT protocol. After 24 h, medium was replaced, and cells were transfected with 2 µg SNAP-KDEL using Lipofectamine 2000 according to the manufacturer’s protocol. Medium was replaced after 6 h, and cells were imaged 24 h later.

### Phenotype criteria for KO/knockdown

WT or Rtn4KO U2OS cells treated with siRNA and transfected with SNAP-KDEL were labeled with SiR-BG before acquiring STED images of live cells. Multiple STED images of sheet-containing subregions of a cell, with a 9.7 × 9.7-µm field of view, were acquired if the cell was labeled with SiR-BG without obvious SNAP-KDEL overexpression artifacts (fluorescent clumps or inflated-looking ER tubules) and if the cell contained a single intact nucleus. To score the phenotypes shown in Fig. 3 C, STED images were evaluated by H. Merta and L.K. Schroeder to first determine whether any subregion of the cell was enriched with sheets. Cells enriched with ER sheets were required to have at least one subregion with a continuous large sheet that spanned one side of the image and with at least one sheet subregion with uniform SNAP-KDEL intensity. Cells were then scored as containing only uniform sheets or containing nonuniform regions that have nanoholes and other structures. Nonuniform sheets fit all of the following criteria: (a) had at least one identifiable hole, (b) had holes where the fluorescence intensity around the hole was less than the intensity at the edge of sheet and with the hole minimum intensity less than sheet intensity, and (c) had at least one hole that was not at the outer edge of a sheet. If the SNAP-KDEL intensity in a cell was too dim to determine whether the sheet contained features, the cell was marked as not scorable.

### Immunofluorescence and antibodies

Cells were fixed and immunolabeled similar to COS-7 ER samples described by Huang et al. (2016). Briefly, cells were fixed with 3% paraformaldehyde (15710; Electron Microscopy Sciences) + 0.1% glutaraldehyde (16019; Electron Microscopy Sciences), permeabilized with 0.3% IGEPAL CA-630 (18896; Sigma-Aldrich) + 0.05% Triton X-100 (T8787; Sigma-Aldrich), and blocked with goat or donkey normal serum (Jackson ImmunoResearch). For Fig. 1 F, goat anti-Rtn4 (Nogo N-18; sc-11027; Santa Cruz Biotechnology) was labeled with custom-labeled secondary antibody: unlabeled donkey anti-goat antibodies (Jackson ImmunoResearch) were labeled per manufacturer’s directions with Atto594 NHS ester (Sigma-Aldrich). Goat anti-Rtn4 was imaged in cells that transiently expressed SNAP-Sec61β, which was labeled with SiR-BG in living cells (see Preparation for live-cell imaging section). For 4Pi-SMS (Fig. 2, E–I) and STED imaging of overexpressed GFP-Sec61β (Fig. S1, B–D), GFP was immunolabeled with rabbit anti-GFP (A-11122; Invitrogen) and a secondary goat anti-rabbit Alexa Fluor 647 (A-21245; Invitrogen) for 4Pi-SMS or Atto594 antibody (77671-1ML-F; Sigma-Aldrich) for STED imaging. Immunoblots were probed with rabbit anti-HaloTag (G9281; Promega) or goat anti-Rtn4 (sc-11027; Santa Cruz Biotechnology).

### Preparation for live-cell imaging

For live-cell imaging, cells were seeded on glass-bottomed dishes (35 mm; no. 1.5; MatTek). Cells expressing SNAP or Halo-tagged proteins were labeled in growth medium immediately before imaging. SiR dye was used to label cells for live-cell STED imaging. SiR-BG (SNAP-cell 647-SiR; S9102S; New England Biolabs) and SiR-CA (gift from K. Johnsson, Max Planck Institute for Medical Research, Heidelberg, Germany; Lukinavičius et al., 2013) were incubated with cells at a concentration of 1 or 0.25 µM, respectively for a minimum of 15 min and up to 1 h at 37°C to label cells. SNAP-Cell Oregon Green (S9014S; New England Biolabs) and SNAP-Cell TMR-Star (S9015S; New England Biolabs) were labeled at 1 µM for confocal imaging. After labeling, cells were rinsed three times with fresh growth medium and then incubated in growth medium for a minimum of 15 min and up to 1 h at 37°C before imaging.

### Preparation for 4Pi-SMS imaging

Coverglass was cleaned by sonication in 1 M KOH, rinsed with water, coated with poly-l-lysine (Sigma-Aldrich), and seeded with COS-7 cells that had been electroporated to express GFP-Sec61β (see Cell culture methods). Cells were immunolabeled with rabbit anti-GFP and goat anti-rabbit Alexa Fluor 647 and then postfixed. Next, 100 nm fluorescent crimson beads (C47248 [discontinued]; Invitrogen) were sparsely added to the top of the cell sample for microscope alignment. Samples were mounted in an aluminum sample frame with thiol-containing buffer (β-mercaptoethanol, catalase, and glucose oxidase diluted in 50 mM Tris, pH 8.0, 50 mM NaCl, and 10% glucose) sandwiched between the cells on coverglass and an additional clean coverglass. Sample frames were sealed using two-component silicone putty (Picodent Twinsil; Picodent).

### Microscopy

To acquire live- and fixed-cell fluorescence microscopy images at ∼50-nm resolution, we used STED microscopy, a superresolution microscopy technique independent of potentially artifact-inducing image-processing algorithms. Living samples were imaged with 5% CO_2_ and heated to 37°C using a stage incubator and objective heater. Cells were imaged in Live Cell Imaging Solution (Invitrogen) supplemented with 15 mM d-glucose. Fixed STED samples were mounted in ProLong Diamond Antifade Mountant (Invitrogen) and imaged at room temperature. Images were acquired if the labeled cell contained a single intact nucleus and did not show any obvious artifacts from overexpressing Halo-KDEL, SNAP-KDEL, or SNAP-Sec61β, which occur in the brightest cells as fluorescent clumps or inflated-looking ER tubules.

STED images were acquired using a Leica SP8 STED 3× equipped with a SuperK Extreme EXW-12 (NKT Photonics) pulsed white light laser as an excitation source and Onefive Katana-08HP pulsed laser as a depletion light source (775-nm wavelength). All images were acquired using a high-contrast Plan Apochromat 100× 1.40 NA oil CS2 objective and Application Suite X software (LAS X; Leica Microsystems). Line scan speed of 8,000 Hz was used for live-cell imaging, and 1,000 Hz was used for fixed-cell imaging. Videos and some images were acquired with bidirectional scanning, which effectively yields 16,000-Hz scan speed. SiR was imaged with 633-nm excitation and 775-nm depletion wavelengths. Atto594 was imaged with 594-nm excitation and 775-nm depletion wavelengths. The depletion laser power was 111 mW at the sample for live and fixed STED images. SiR fluorescence emission light between 650 and 750 nm was collected with a HyD hybrid detector. Oregon Green was imaged with 488-nm excitation, and 500–575-nm fluorescence was collected with a HyD hybrid detector. TMR was imaged with 554-nm excitation, and 580–620-nm fluorescence was detected with a photomultiplier tube. The detection gate was set at 0.3–6 ns during STED imaging. The pinhole size was set to 1 Airy unit.

Images were acquired with 1,024-, 512-, or 256-pixel square fields of view, with the exception of videos in which a 256 × 64-pixel field of view was used. All STED images had 18.9-nm pixel size. One-color live-cell videos were acquired with 70 ms per frame. The line sequential imaging mode was used for live-cell two- and three-color imaging to avoid channel synchronization artifacts. All live-cell images were acquired using 16 line averages. Fixed images of NE and immunolabeled RTN4 were acquired with 32 line averages.

To acquire fixed-cell fluorescence microscopy images at 20-nm x, y, and z resolution, we used 4Pi-SMSN. Fixed one-color 4Pi-SMSN samples labeled with Alexa Fluor 647 were imaged as previously described (Huang et al., 2016). Briefly, the fluorescence emission was coherently collected by two opposing objectives (100×/1.35, silicone oil immersion; Olympus) and imaged on a sCMOS camera (ORCA-Flash 4.0 V2; Hamamatsu Photonics). The samples were imaged at room temperature on the 4Pi microscope at 200 fps with an excitation laser (642 nm) intensity of ∼15 kW/cm^2^. 4Pi image analysis was done as previously described (Huang et al., 2016). Custom-written LabVIEW software was used for acquisition. iSIM videos were acquired using a VT-iSIM (VisiTech; courtesy of BioVision Technologies) at room temperature and 15-ms frame acquisition times.

### Image processing

Images were visualized, smoothed, and contrast-adjusted using ImageJ (National Institutes of Health) as described below. Although displayed images are smoothed, all analysis was performed on raw data. STED and confocal images were smoothed for display with a 1- and 3-pixel sigma Gaussian blur, respectively, when using 18.9-nm pixel size. Low-magnification confocal images were smoothed with a 1-pixel sigma Gaussian blur. Minimum and maximum brightness were adjusted linearly. Videos were bleach corrected using histogram matching. Manual tracking with local minimum center correction was used to follow ER hole movement. For 4Pi-SMSN images, custom-written MatLab code was used to localize blinking fluorescently labeled molecules. Vutara SRX software was used for visualization.

### Data analysis

To measure tubule diameters, 10-pixel-wide and at least 700-nm-long line profiles were manually drawn perpendicular to the long axis of an ER tubule using ImageJ. Lines were drawn perpendicular to straight regions of tubules. Fluorescence intensity and coordinate arrays were exported to Excel and saved for further analysis. We determined ER tubule diameters by fitting individual fluorescence intensity line profiles of labeled tubules to a function that combines the expected location of the dye and the PSF of the microscope. Line profiles were fitted with a projection of either an idealized surface-labeled cylinder or label-filled cylinder convolved with a Lorentzian PSF, iteratively changing both the tubule diameter and PSF FWHM values in a nested loop to find the best fit. We report diameter and PSF FWHM values that give the best fit by minimizing the squared error. For SNAP-Sec61β–labeled membranes, we modeled the distribution of dyes as a uniform 4.5-nm-thick annulus, which accounts for the size of the SNAP-tag, and for GFP-Sec61β–labeled membranes, we modeled the dye distribution as a uniform 17.5-nm-thick annulus to account for the size of primary and secondary antibodies (Weber et al., 1978; Barentine et al., 2018). Tubule diameter values measure the distance between the outer leaflets on either side of surface-labeled ER tubules or the distance between the inner leaflets for label-filled ER tubules. Tubule fitting and determination of the microscope PSF was performed using the PYthon-Microscopy Environment (PYME) and the NEP fitting plugin described by Barentine et al. (2018). Because membrane and lumen-labeled tubule line profiles were fitted with different functions, the PSF widths used in tubule diameter measurements were optimized separately and yielded 50.9 nm (*n* = 6 cells; *n* = 3 d) for membrane-labeled and 45.8 nm (*n* = 8 cells; *n* = 3 d) for lumen-labeled cells. For Fig. 2 A, the PSF fit was run separately for tubules from different cells on different days. We found that changing the PSF FWHM to other values within the range of per-cell PSF measurements (for all 49.3 ± 5.0 nm, mean ± SD; for membrane-labeled 53.0 ± 4.3 nm, mean ± SD; and for lumen-labeled 46.5 ± 3.6 nm, mean ± SD) does not have a substantial effect on the tubule diameter measurements.

Image brightness as shown in Fig. 2 (K–N) was analyzed with 5-pixel-wide line profiles. Data points are shown in Fig. 2 (L and N) as intensity values from the raw image, whereas the fluorescence intensity line profile shows data from the same image extracted after smoothing with a 3 × 3-pixel uniform filter. Because our xy resolution (∼50 nm) is roughly half the diameter of an ER tubule (Fig. 2, A and D), we can resolve intensity changes across the diameter of tubules. Our z resolution (∼600 nm) is sufficiently large to sample the full height of ER tubules or ER sheet structures. Thus, the intensity of the ER image allows us to distinguish thicker and brighter ER tubules that are ∼100 nm in diameter from thinner and dimmer ER sheets that are 30–50 nm in thickness, assuming that the marker is evenly distributed throughout the ER lumen.

To measure holes (Fig. 4, D and E; and Fig. S3, A–F), holes in an ER sheet image were manually identified, only excluding large holes that clearly represent large loops of tubules several hundred nanometers in diameter that are not holes in sheets. Holes, which include nanoholes as a subset, were defined as regions in ER-labeled cells that contain several dim pixels surrounded by brighter fluorescent regions. We specifically define nanoholes as those dim-pixel holes within uniformly labeled ER sheets (example of uniform sheet intensity shown as green bars in Fig. 2, K–N), with hole boundaries >100 nm in the xy plane (mean thickness of an ER tubule; Fig. 2 D), and easily distinguishable from dense tubular networks based on their fluorescence intensity profiles (see Fig. S2, F–I). We use this nanohole definition throughout this study. Live images of unperturbed, GFP-Climp63 overexpressing, and nocodazole-treated cells were analyzed. Fixed cells expressing GFP-Sec61β for 48 h were immunolabeled, and their images were used to measure holes in the NE where NPCs reside. Fixed immunolabeled NE images were used for measuring holes because these samples had better signal-to-noise ratios than live images of the NE.

Hole boundary detection and characterization were performed using code written in Python. Segmentation and smoothing were performed using recipe modules from PYME. The data were smoothed slightly with a Gaussian filter with a kernel width of σ = 1 pixel. Hole candidates were identified by searching for local minima with a minimum separation of 4 pixels. A binary image of the ER was generated by first max-normalizing the data, applying a median filter with a 15 × 15-pixel kernel, and thresholding at 5% of the maximum value. Segmentation was then performed on the masked and smoothed data using a watershed algorithm with the hole candidates as the markers. The raw images, not the segmented regions, were displayed to L.K. Schroeder, S. Bahmanyar, and A.E.S. Barentine, who manually identified the approximate center of each hole in the raw data, establishing which watershed regions the program would then analyze. Within these watershed regions, a hole border was defined using a functionally agnostic approach. This was accomplished by first determining the hole depth as the difference between the mean of the pixels comprising the edge of the watershed region on the smoothed image and the minimum pixel value within the region on the smoothed image. The pixels below the minimum pixel value plus 40% of the hole depth were determined to be within the hole, and the raw data corresponding with these points were used for further quantification. Poorly segmented holes were rejected manually.

The hole symmetry and equivalent diameter of each hole were calculated using code written in Python. The symmetry was determined by first constructing a matrix of the x and y coordinates of each pixel inside of the hole. For an elliptical hole whose major and minor axes are parallel to the Cartesian axes, our approach calculates the SD of the pixel positions along the minor axis and divides that by the SD along the major axis. To determine the hole symmetry for arbitrarily shaped holes at various orientations, we calculated the covariance matrix of the pixel position matrix. Because this covariance matrix is by definition symmetric and filled with real values, its diagonalization yields two eigenvectors, one of which points along the direction with the largest variance, and a second that is exactly perpendicular to the first. The eigenvalues for each of these eigenvectors is the covariance along that axis, and we define the hole symmetry as

$$\frac{\sqrt{\lambda_{2}}\;}{\sqrt{\lambda_{1}}},$$

where $\lambda_{1}$ is the larger covariance eigenvalue and $\lambda_{2}$ is the smaller covariance eigenvalue. The equivalent diameter was calculated by assuming the geometry of a circle such that $d\;=\;2\sqrt{A/\pi }$, where *d* is the equivalent diameter and *A* is the area of a pixel multiplied by the number of pixels in a hole. The equivalent diameter and hole symmetry swarm plots were generated using the Seaborn Python package.

### Nanohole simulation

Images of nanoholes were numerically simulated in a custom Python script. Holes were modeled as they were in the geometric model: cylindrical holes in a 50-nm-thick sheet, with curved inner edges defined by the inner half of a torus. Four images of 25 holes each, spaced by 105 pixels (curved edge to curved edge), were simulated with 30-, 50-, 75-, 100-, 125-, 150-, and 200-nm inner diameters. Hole centers were jittered using a uniform distribution to eliminate artifacts from holes always being centered on pixels. To mimic imaging with our STED microscope, the lateral pixel size was simulated to be 18.9 nm, the model was convolved with a 50-nm FWHM Lorentzian PSFw (to emulate the PSF of the STED microscope), and the convolved model was used as expected values for sampling Poisson distributions (to add shot noise). The model was first generated in three dimensions with an axial pixel size of 1 nm, and each voxel determined to be luminal by the model was filled with a value of α, which is analogous to fluorescence intensity per unit volume. The model was then projected onto the lateral plane before numeric convolution. Noise was added similar to that observed in our live-cell images; this was accomplished by adding a background of 1 and setting α to 10 for generating the preconvolved model. For measurements shown in Fig. S3 (A–D), the hole images were processed with the same algorithm used for analyzing our live-cell images, and the holes were manually identified by A.E.S. Barentine. After the convolution and addition of shot noise, many of the 30-nm holes were unidentifiable and were therefore not selected for analysis. Swarm plots were generated using the Seaborn Python package.

### Statistical analysis

Prism7 (GraphPad Software) was used to evaluate the statistical significance of hole symmetry and equivalent hole diameter. The distributions of our hole measurements do not appear Gaussian. We therefore used the nonparametric Kruskal–Wallis test with a post hoc Dunn’s test for multiple comparisons in Fig. 4 (D and E).

### Analytic ER modeling

To determine how different ER sheet thicknesses affect the curved surface area of the ER as shown in Fig. 5, we modeled uniform sheets, tubules, and nanoholes using geometric shapes. Our model of the ER comprises a laterally oriented disc to describe a sheet, a laterally oriented cylinder to describe a tubule, empty axially oriented cylinders to describe nanoholes, and toroid inner and outer halves to define curved edges of holes and sheets. We restricted our modeling to ER morphologies attainable without lipid addition or subtraction by keeping the surface area constant to that of a 5-µm-diameter sheet of a given thickness without a tubule or holes, which is the surface area that all normalizations were performed with respect to. To keep total surface area constant, the diameter of the sheet was varied depending on the number of holes or length of tubule being modeled. Both tubules and holes were modeled to consist entirely of positive curvature; however, holes have saddle-type curvature that includes additional negative curvature along the axis orthogonal to the positive curvature axis. We report the area of curvature and do not distinguish between positive curvature that had or lacked additional orthogonal negative curvature. We calculated the curved membrane surface area as a fraction of the total surface area (which was fixed) as 100-nm-inner-diameter nanoholes (Fig. 4 E) were inserted, and lengths of 100-nm-diameter tubule (Fig. 2 D) were extended from a 100-nm, 50-nm-thick sheet, and 30-nm-thick sheet. For all three sheet thicknesses, we calculated the fraction of ER surface area that was curved as the number of 100-nm-inner-diameter nanoholes and length of 100-nm-diameter tubule were both varied. The resulting isoclines of constant curved fraction of surface area were nearly linear, and the linear approximations were used to generate Fig. 5. Our ER model was composed in Mathematica.

### Online supplemental material

Fig. S1 shows ER nanoholes marked by a membrane-localized ER marker, NE holes colocalized with Nup160-Halo^EN^, localization of Rtn4^EN^-SNAP to nanoholes in flat ER sheets, and ER sheets visualized by iSIM. Fig. S2 shows plots of ER tubule diameters measured with luminal ER markers, plots of simulated sheet thickness, and representative fluorescence intensity profiles of ER sheets with nanoholes versus tubule matrices. Fig. S2 also shows characterization of the Rtn4 KO U2 OS cell line and ER phenotypes in Rtn4 KO cells codepleted for Rtn1/3. Fig. S3 shows plots of hole symmetry and equivalent diameters from simulated holes of different sizes, a representative gallery of holes, the watershed algorithm applied to define hole edges for analysis, and localization of Rtn4^EN^-SNAP under different conditions related to Fig. 4. Video 1 is related to Fig. 1 (H and I) and shows a time-lapse of ER nanohole dynamics. Video 2 is related to Fig. S1 I and shows an iSIM video of ER sheet. Video 3 is related to Fig. 4 (F and G) and shows a time-lapse of ER nanohole dynamics in a GFP-Climp63–overexpressing cell. Table S1 is a resource table of all reagents used in this study. Text S1 contains the Mathematica script for the ER model related to Fig. 5.

## Supplementary Material

Supplemental Materials (PDF)

Text S1 (PDF)

Video 1

Video 2

Video 3
