# Supplementary material for: Dynamic nanoscale morphology of the ER surveyed by STED microscopy
Source: J Cell Biol. 2019 Jan 7;218(1):83–96. doi: 10.1083/jcb.201809107 (PMC6314542; doi:10.1083/jcb.201809107)
Supplement: Supplemental Materials (PDF) [file JCB_201809107_sm.pdf]

JCB

# Supplemental material

Schroeder et al., https://doi.org/10.1083/jcb.201809107

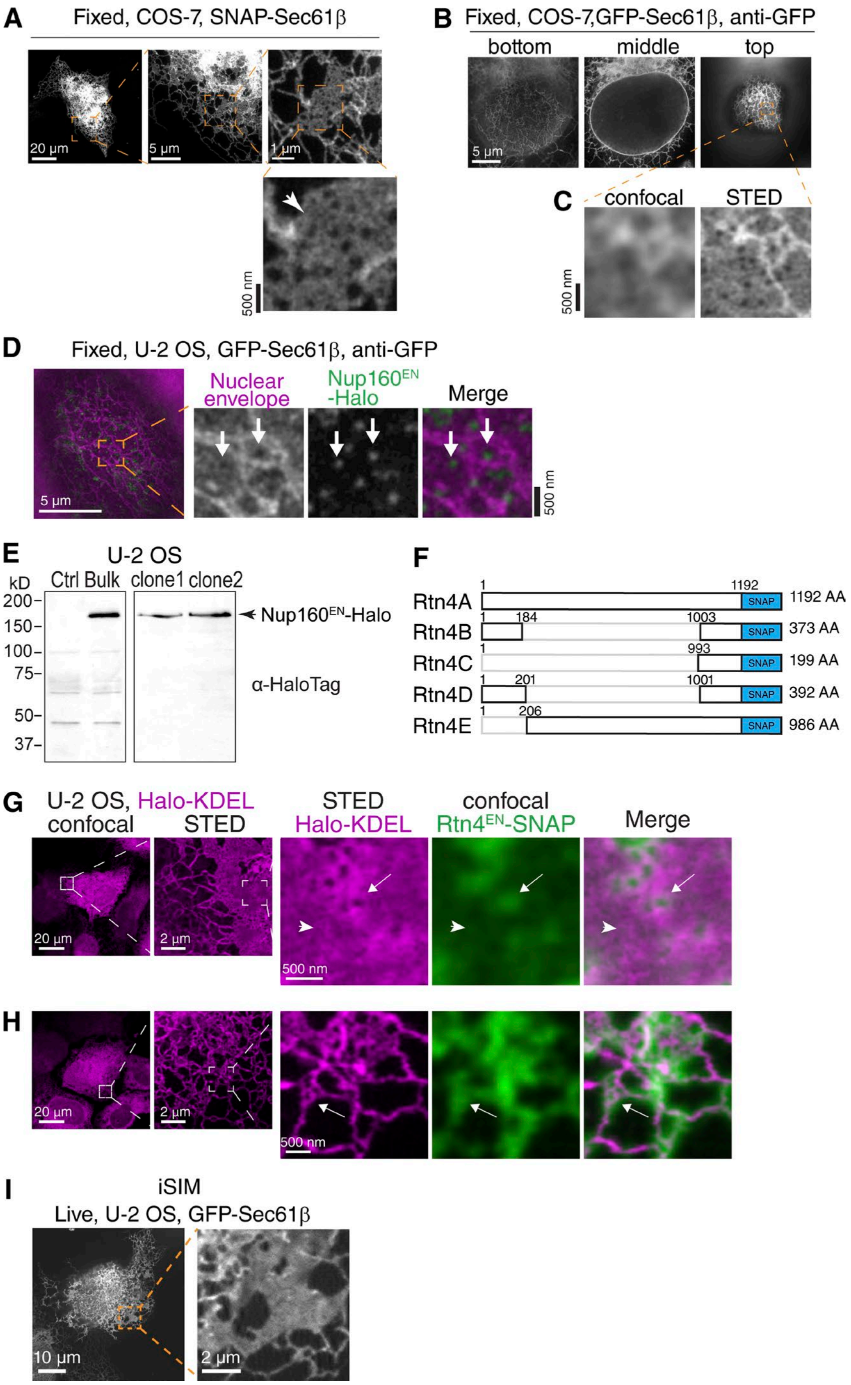


Figure S1. **STED images of membrane-labeled nanoholes in ER sheets and NE holes, colocalization of Nup160$^{EN}$-Halo to NE holes, and Rtn4$^{EN}$-SNAP at ER nanostructures. (A)** Overview confocal and magnified STED image of fixed COS-7 cell expressing SNAP-SEC61β labeled with SiR-BG. Nanoholes can be seen in peripheral ER sheets using SNAP-SEC61β, which labels the cytosolic face of the ER membrane. **(B)** STED images of bottom, middle, and top of the NE of a fixed COS-7 cell expressing GFP-SEC61β. **(C)** Magnified confocal and STED images of the dashed box region from the top image in B. **(D)** STED images of a fixed CRISPR-Cas9 genome-edited U2OS cell expressing Nup160 tagged with HaloTag at the endogenous locus (Nup160$^{EN}$-Halo; green in merge) and expressing GFP-SEC61β immunolabeled with anti-GFP antibodies (magenta in merge). Arrows in magnified images mark holes in the NE occupied by Nup160. **(E)** Immunoblot of WT (Ctrl) and genome-edited Nup160$^{EN}$-Halo bulk and clonal populations probed with anti-HaloTag antibody. Clone 2 was used in D. **(F)** Schematic of Rtn4 isoforms as indicated in PubMed (Gene) and Ref-Seqs (adapted from Grumati et al., 2017) fused to SNAP tag in endogenously tagged Rtn4-SNAP$^{EN}$ cells. **(G and I)** Overview of confocal and magnified STED images of Halo-KDEL in living U2OS cells (magenta). Line sequential confocal images of Rtn4-SNAP$^{EN}$ (green). Arrowhead in G shows uniform ER sheet devoid of Rtn4-SNAP$^{EN}$, and arrow points to Rtn4-SNAP$^{EN}$ localization to nanoholes. Rtn4-SNAP$^{EN}$ (green) localizes throughout tubular network (magenta) in H. **(I)** iSIM image of U2OS cells stably expressing GFP-SEC61β. Left panel shows overview of ER network and a peripheral ER sheet marked with a dashed box, which is magnified in the right panel. Region shown in right panel is also shown in Video 2.

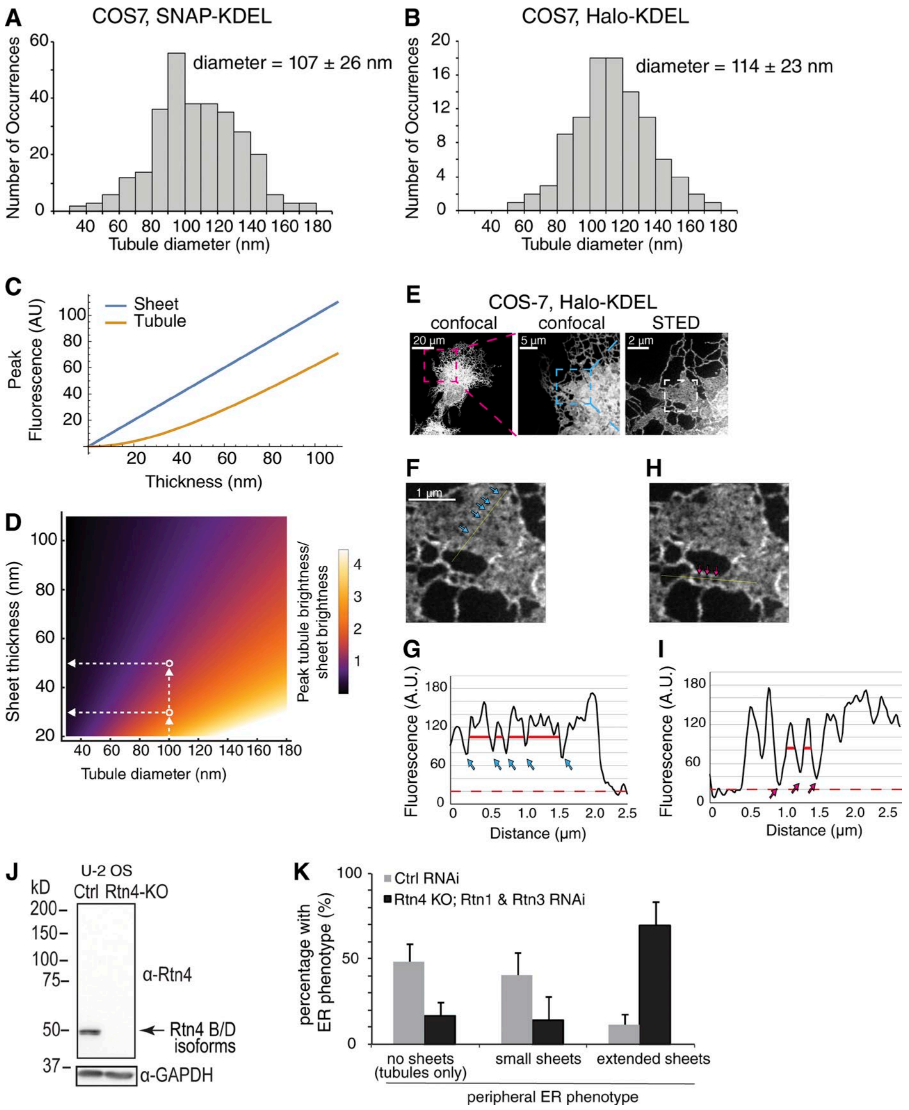


Figure S2. **Tubule diameters of luminal labeled ER tubules, simulated peak brightness of sheets and tubules, intensity profiles of ER nanostructures, and characterization of genome-edited Rtn4-KO cell line. (A)** Histogram of ER tubule diameters measured from live-cell STED images of SNAP-KDEL–expressing COS-7 cells (*n* = 300 tubules; *n* = 3 imaging days; diameter = mean ± SD). The microscope resolution by NEP-fitted PSF FWHM is 45.8 nm. **(B)** A histogram of ER tubule diameters measured from live-cell STED images of Halo-KDEL–expressing COS-7 cells (*n* = 100 tubules; *n* = 1 imaging day; diameter = mean ± SD). The microscope resolution by NEP-fitted PSF FWHM is 46.6 nm. The small difference in the average diameter values between membrane and lumen-labeled tubules may derive from nonuniform luminal distribution of KDEL (e.g., binding to membrane-anchored KDEL receptor). As previously shown (Barentine et al., 2018), membrane-labeled tubules result in better separation of diameter estimates from microscope resolution, leading to the conclusion that the membrane-labeled values are more robust. **(C)** Plot of peak fluorescence intensity as simulated ER sheet thickness and tubule diameter change. The STED PSF is assumed to be Lorentzian, with 50 nm FWHM in the xy plane and a z FWHM larger than both ER sheet and tubule thicknesses such that the z axis can be summed. The peak fluorescence of a sheet is linearly dependent on sheet thickness and is not affected by the convolution inherent to the imaging process, since it is surrounded by more sheet. The peak fluorescence of a tubule, however, would depend linearly on the thickness of the tubule, but is decreased by the convolution, since it is assumed not to be close to other structures. **(D)** Heatmap showing the fraction of peak tubule brightness divided by sheet brightness for various sheet thicknesses and tubule diameters. Two points are outlined in white, which highlight that this fraction is 1.24 for a 50-nm-thick sheet and 100-nm-diameter tubule, and 2.06 for a 30-nm-thick sheet and 100-nm-diameter tubule. **(E)** Confocal and STED images of Halo-KDEL in living COS-7 cells shown in Fig. 1 (A–D). Dashed box region in right panel shown in F and H. **(F–I)** Yellow line in F and H represents 1-pixel-wide fluorescence intensity line profile shown in G and I from a STED image smoothed with a 1-pixel Gaussian blur. Cyan arrows mark nanoholes in a uniform sheet, whose minimum intensities are well above background level, marked with a dashed red line, indicating the small size of nanoholes. The distance between nanoholes, length of solid red line, varies over a wide range and is usually larger than the diameter of a tubule. Magenta arrows mark holes in a dense tubule network, whose minimum fluorescent intensities are close to background level, marked with red dashed line. The distance between holes in a dense tubule network is approximately equal to the diameter of a tubule. **(J)** Immunoblot of control and CRISPR-Cas9 genome-edited Rtn4-KO U2OS cells. The Rtn4 antibody that was used throughout this study recognizes Rtn4 A/B/D isoforms, and identified the Rtn4 B/D isoforms are expressed in WT U2OS cells. **(K)** Characterization of ER phenotypes observed by confocal microscopy in WT and Rtn4 KO U2OS cells treated with control and Rtn1/Rtn3 siRNA, respectively. To score phenotypes, confocal images were evaluated as having (1) tubules only, (2) tubules and small sheets, or (3) tubules, small sheets, and large sheets (tubules only, small sheets, and extended sheets, respectively). *n* = 5 d; *n* = 265 cells. Error bars show standard deviation.

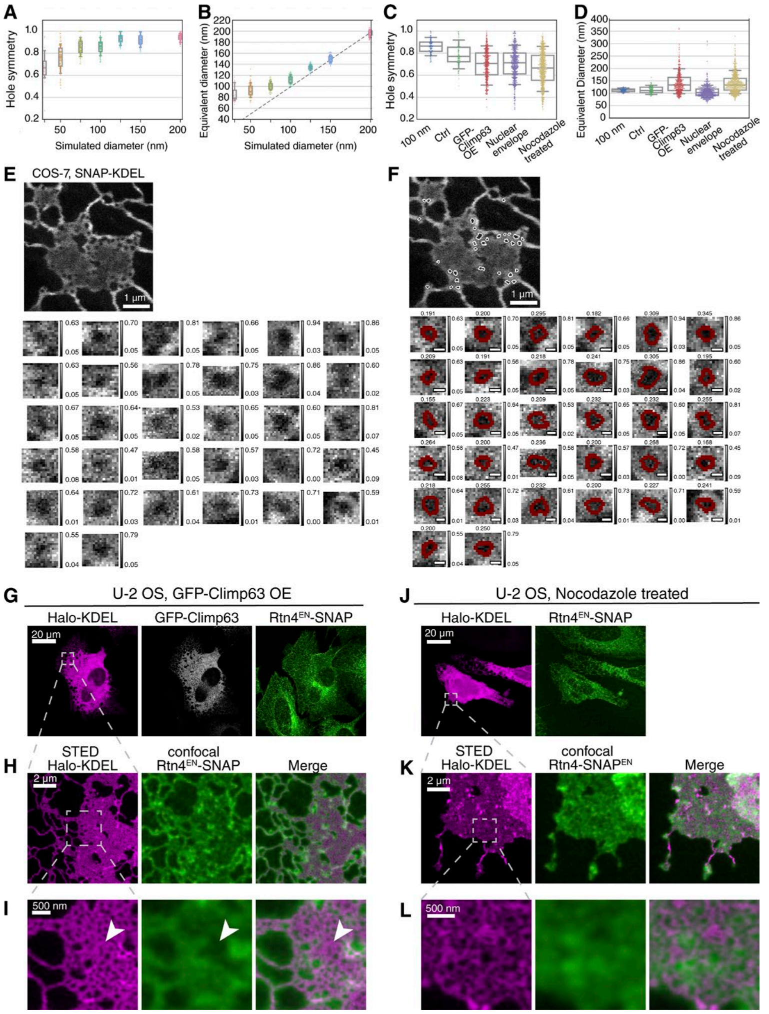


Figure S3. **Measurements of simulated nanohole images with varied diameters, gallery of identified holes and boundaries, and Rtn4$^{EN}$-SNAP localization to ER nanostructures under different conditions. (A)** Plot of hole symmetry from simulated images of nanoholes with 30-, 50-, 75-, 100-, 125-, 150-, and 200-nm inner hole diameter, referred to as "diameter," ($n$ = 22 for 30-nm diameter; $n$ = 100 for all others). Hole symmetry is determined by calculating the covariance matrix of the xy positions of each pixel in a nanohole, and then taking the fraction of the square root of the smallest covariance eigenvalue divided by the square root of the remaining (orthogonal) covariance eigenvalue. Although nanoholes were modeled as circles (symmetry = 1), the simulated images may appear elongated due to added Poisson noise and the center location; this is most pronounced for small nanoholes with diameters equal to or below the resolution of the simulated microscope (50 nm PSF FWHM). **(B)** Plot of measured equivalent diameter from simulated images of nanoholes with 30-, 50-, 75-, 100-, 125-, 150-, and 200-nm diameter ($n$ = 22 for 30 nm diameter; $n$ = 100 for all others). The equivalent diameter is the diameter of a nanohole based on its area if the hole were assumed to be perfectly circular. Median equivalent diameter values for 30-, 50-, 75-, 100-, 125-, 150-, and 200-nm diameters were 78, 90, 100, 113, 133, 149, and 198 nm, respectively. The equivalent diameter accurately describes the actual diameter of nanoholes with ≥100 nm diameters. Gray dashed line plots equal simulated diameter and measured equivalent diameter. **(C)** Plot of hole symmetry from Fig. 4 D with added measurements from simulated images of 100-nm-diameter nanoholes. These simulated nanoholes are more symmetric than all holes measured from images of cells. When ER sheets or the NE is tilted relative to the focal plane, perfectly circular holes will have symmetry values <1. Simulated holes were modeled to be perfectly flat and their hole symmetry values are closer to 1. The tilt effect has a cosθ dependence, where θ is 0 if the hole is flat, and is therefore relatively muted for reasonable tilt angles. With this in mind, noise is likely the larger factor introducing asymmetry. **(D)** Plot of equivalent diameter from Fig. 4 E with added measurements from simulated images of 100-nm-diameter nanoholes. These simulated nanoholes are measured by our algorithm to have slightly >100-nm diameters, similar to holes in the NE. We expect that our edge-finding algorithm and diameter measurement overestimates the NE hole equivalent diameters by ∼10%. Tilt in the ER or NE membrane relative to the focal plane would make holes appear smaller; however, this effect again has a cosθ dependence, where θ is 0 if the hole is flat and would therefore take a substantial tilt to see an effect. For all plots, the median and interquartile range is shown, and whiskers are drawn down to the 10th percentile and up to the 90th percentile. **(E)** Image and hole gallery of all holes manually targeted for automatic analysis from a single image of ER sheet in an unperturbed cell. The dimensions of each cropped hole region vary, but all have 18.9-nm pixel size. The pixel intensity values of the entire image were min/max normalized before hole regions were cropped. The displayed color map for each cropped hole image is self-normalized (min/max) to better show the hole boundaries. **(F)** Image and hole gallery in E with autosegmented hole boundaries shown in white for overview image and autosegmented hole boundaries shown in red for individual holes. Bars, 100 nm. **(G–I)** Overview confocal and STED images of genome-edited U2OS cells expressing Rtn4-SNAP$^{EN}$ from the endogenous locus (green) and transiently transfected with GFP-Climp63 (grayscale) and Halo-KDEL (magenta). Nanoholes are present throughout sheets in Climp63-overexpressing (OE) cells. Rtn4$^{EN}$-SNAP was excluded from uniform sheets, which are occasionally present in Climp63-overexpressing cells (arrowheads). **(J–L)** Overview confocal and STED images of genome-edited U2OS cells expressing Rtn4-SNAP$^{EN}$ from the endogenous locus (green) and transiently transfected with Halo-KDEL (magenta) treated with 33 µM nocodazole to depolymerize microtubules.

Table S1. **Key resources**

| Reagent or resource | Source | Identifier |
|---|---|---|
| COS-7 | ATCC | CRL-1651 |
| U2OS | ATCC | HTB-96 |
| Halo-KDEL | This work | |
| SNAP-KDEL | This work | |
| Halo-Sec61β | Bottanelli et al. (2016) | |
| SNAP-Sec61β | Bottanelli et al. (2016) | |
| GFP-Sec61β (mEmerald-Sec61-C-18) | Addgene | 54249 |
| AcGFP1-MsClimp63 | Shibata et al. (2010) | |
| Goat anti-Nogo (N-18) | Santa Cruz Biotechnology | sc-11027 |
| Rabbit anti-HaloTag | Promega | G9281 |
| Rabbit anti-GFP | Thermo Fisher Scientific | A-11122 |
| SNAP-Cell 647-SiR (SiR-BG) | New England Biolabs | S9102S |
| SNAP-Cell TMR | New England Biolabs | S9015S |
| SNAP-Cell Oregon Green | New England Biolabs | S9014S |
| SiR-CA | Lukinavičius et al. (2013) | |
| pSpCas9 (PX165) | Addgene | 48137 |
| pSPCas9(BB)-2A-Puro (PX459) v2.0 | Addgene | 62988 |
| pEGFP-N1 | Takara Bio Inc. | 6085-1 |
| Silencer Select Negative Control siRNA 1 | Ambion | 4390843 |
| Silencer Select RTN1 siRNA | Ambion | 4392420-S12378 |
| Silencer Select RTN3 siRNA | Ambion | 4392420-S20162 |

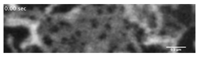

Video 1. **70-ms/frame STED video of nanoholes in ER sheet.** Related to Fig. 1 (H and I). COS-7 cell expressing SNAP-KDEL labeled with SiR-dye. One STED image was acquired in 0.07 s with a total of 10 consecutive frames shown. Video is smoothed with 1-pixel sigma Gaussian blur, bleach corrected, and shown at 2 frames/s.

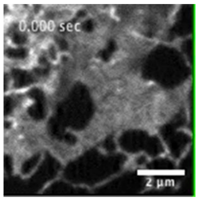


Video 2. **15-ms/frame iSIM video of ER sheet.** Related to Fig. 1 I. U2OS cell stably expressing GFP-Sec61β. iSIM video with 0.015 s per frame for a total duration of 12 s. Video is shown at 66 frames/s in real time.

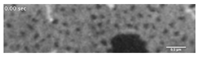

Video 3. **70 ms/frame STED video of an ER sheet in a cell overexpressing GFP-Climp63.** Related to Fig. 4 (F and G). COS-7 cell expressing SNAP-KDEL labeled with SiR and GFP-Climp63 (see Fig. 4 F for image of GFP). One STED image was acquired in 0.07 s with a total of 10 consecutive frames shown. Video is smoothed with 1-pixel sigma Gaussian blur, bleach corrected, and shown at 2 frames/s.

**Text S1 is a separate PDF containing the Mathematica script for the ER model related to Fig. 5.**

## References

Barentine, A.E.S., L.K. Schroeder, M. Graff, D. Baddeley, and J. Bewersdorf. 2018. Simultaneously Measuring Image Features and Resolution in Live-Cell STED Images. *Biophys. J.* 115:951–956. https://doi.org/10.1016/j.bpj.2018.07.028

Bottanelli, F., E.B. Kromann, E.S. Allgeyer, R.S. Erdmann, S. Wood Baguley, G. Sirinakis, A. Schepartz, D. Baddeley, D.K. Toomre, J.E. Rothman, and J. Bewersdorf. 2016. Two-colour live-cell nanoscale imaging of intracellular targets. *Nat. Commun.* 7:10778. https://doi.org/10.1038/ncomms10778

Grumati, P., G. Morozzi, S. Hölper, M. Mari, M.I. Harwardt, R. Yan, S. Müller, F. Reggiori, M. Heilemann, and I. Dikic. 2017. Full length RTN3 regulates turnover of tubular endoplasmic reticulum via selective autophagy. *eLife.* 6:e25555. https://doi.org/10.7554/eLife.25555

Lukinavičius, G., K. Umezawa, N. Olivier, A. Honigmann, G. Yang, T. Plass, V. Mueller, L. Reymond, I.R. Corrêa Jr., Z.G. Luo, et al. 2013. A near-infrared fluorophore for live-cell super-resolution microscopy of cellular proteins. *Nat. Chem.* 5:132–139. https://doi.org/10.1038/nchem.1546

Shibata, Y., T. Shemesh, W.A. Prinz, A.F. Palazzo, M.M. Kozlov, and T.A. Rapoport. 2010. Mechanisms determining the morphology of the peripheral ER. *Cell.* 143:774–788. https://doi.org/10.1016/j.cell.2010.11.007
