# Supplementary material for: Dynamic nanoscale morphology of the ER surveyed by STED microscopy
Source: J Cell Biol. 2019 Jan 7;218(1):83–96. doi: 10.1083/jcb.201809107 (PMC6314542; doi:10.1083/jcb.201809107)
Supplement: Text S1 (PDF) [file JCB_201809107_TextS1.pdf]

ER Model

Definitions:

Holes

The holes are assumed to be circular, with a curved edge, i.e. a cylinder is removed, and a half torus is added.

Pappus's centroid theorem: surface area is the product of the arc length of the surface multiplied by the distance traveled by its geometric centroid. This means that defining the surface area of a hole as half of a toroid, we can't just use half of the toroid surface area, because the geometric centroid travels a different arc-length.

The second theorem of Pappus's centroid theorem states that volume is product of surface area times the distance traveled by the geometric centroid.

Note that a half circle has a centroid of $\frac{4r}{3\pi}$

Here, bigR is the radius of the flat part that is cut out of the sheet, and little r is half the thickness of the sheet

$$\text{halfTorusSA[bigR\_, r\_]} = (\pi * r) * \left(2\pi * \left(\text{bigR} - \frac{4 * r}{3\pi}\right)\right);$$

$$\text{halfTorusVol[bigR\_, r\_]} = \left(\frac{\pi * r^2}{2}\right) * \left(2\pi * \left(\text{bigR} - \frac{4 * r}{3\pi}\right)\right);$$

The next two definitions apply when considering the outer half of the torus

$$\text{outerHalfTorusSA[bigR\_, r\_]} = (\pi * r) * \left(2\pi * \left(\text{bigR} + \frac{4 * r}{3\pi}\right)\right);$$

$$\text{outerHalfTorusVol[bigR\_, r\_]} = \left(\frac{\pi * r^2}{2}\right) * \left(2\pi * \left(\text{bigR} + \frac{4 * r}{3\pi}\right)\right);$$

If we add a hole to a sheet, the change in surface area is minus two circles of radius bigR and plus one half torus

$$\delta\text{SAhole[bigR\_, thickness\_]} = \text{halfTorusSA}\left[\text{bigR}, \frac{\text{thickness}}{2}\right] - 2 * \left(\pi * \text{bigR}^2\right)$$

$$-2\,\text{bigR}^2\,\pi + \pi^2\,\text{thickness}\left(\text{bigR} - \frac{2\,\text{thickness}}{3\pi}\right)$$

The change in volume for a hole would be minus the volume of the half toroid minus the volume of a

cylinder of height = thickness.

```
δVhole[bigR_, thickness_] = halfTorusVol[bigR, thickness/2] - (thickness * π * bigR^2)
```

$$-\mathrm{bigR}^2\,\pi\,\mathrm{thickness} + \frac{1}{4}\pi^2\,\mathrm{thickness}^2\left(\mathrm{bigR} - \frac{2\,\mathrm{thickness}}{3\,\pi}\right)$$

```
δCurvatureHole[bigR_, thickness_] = halfTorusSA[bigR, thickness/2]
```

$$\pi^2\,\mathrm{thickness}\left(\mathrm{bigR} - \frac{2\,\mathrm{thickness}}{3\,\pi}\right)$$

The Tubule

Let’s ignore the curvature of where the tubule exits the sheet. This allows us to only add one tubule, of varying length, rather than needing to know the average length of a tubule, which would be tough to define. Assume then, that surface area detracted from the sheet by merging into a tubule is exactly equal to the surface area of the cap of the tubule.

```
δSAtub[rTub_, length_] = 2 π * rTub * length (*+ 2π*rTub^2*)
```

$$2\,\mathrm{length}\,\pi\,\mathrm{rTub}$$

```
δVtub[rTub_, length_] = π * rTub^2 * length
```

$$\mathrm{length}\,\pi\,\mathrm{rTub}^2$$

```
δCurvatureTubule[rTube_, length_] = δSAtub[rTube, length];
```

The un-holey sheet

Define the sheet radius as going to the very edge of the flat part of the sheet (leaving the rounded bit out of the radius definition).

```
SAunholey[sheetRad_, thickness_] =
  2 π * (sheetRad)^2 + outerHalfTorusSA[sheetRad, thickness/2]
```

$$2\,\pi\,\mathrm{sheetRad}^2 + \pi^2\,\mathrm{thickness}\left(\mathrm{sheetRad} + \frac{2\,\mathrm{thickness}}{3\,\pi}\right)$$

```
Volunholey[sheetRad_, thickness_] =
  thickness * π * (sheetRad )^2 + outerHalfTorusVol[sheetRad, thickness/2]
```

$$\pi\,\mathrm{sheetRad}^2\,\mathrm{thickness} + \frac{1}{4}\pi^2\,\mathrm{thickness}^2\left(\mathrm{sheetRad} + \frac{2\,\mathrm{thickness}}{3\,\pi}\right)$$

```
CurvatureUnholey[sheetRad_, thickness_] = outerHalfTorusSA[sheetRad, thickness/2]
```

$$\pi^2 \text{ thickness} \left(\text{sheetRad} + \frac{2 \text{ thickness}}{3 \pi}\right)$$

All together now

```
totVolume[initialSheetRad_, thickness_, tubuleRad_, holeRad_,
  tubuleLength_, nHoles_] = Volunholey[initialSheetRad, thickness] +
  δVtub[tubuleRad, tubuleLength] + nHoles * δVhole[holeRad, thickness]
```

$$\text{initialSheetRad}^2 \pi \text{ thickness} + \frac{1}{4} \pi^2 \text{ thickness}^2 \left(\text{initialSheetRad} + \frac{2 \text{ thickness}}{3 \pi}\right) + \text{nHoles} \left(-\text{holeRad}^2 \pi \text{ thickness} + \frac{1}{4} \pi^2 \text{ thickness}^2 \left(\text{holeRad} - \frac{2 \text{ thickness}}{3 \pi}\right)\right) + \pi \text{ tubuleLength tubuleRad}^2$$

```
totSurfaceArea[initialSheetRad_, thickness_, tubuleRad_, holeRad_,
  tubuleLength_, nHoles_] = SAunholey[initialSheetRad, thickness] +
  δSAtub[tubuleRad, tubuleLength] + nHoles * δSAhole[holeRad, thickness]
```

$$2 \text{ initialSheetRad}^2 \pi + \pi^2 \text{ thickness} \left(\text{initialSheetRad} + \frac{2 \text{ thickness}}{3 \pi}\right) + \text{nHoles} \left(-2 \text{ holeRad}^2 \pi + \pi^2 \text{ thickness} \left(\text{holeRad} - \frac{2 \text{ thickness}}{3 \pi}\right)\right) + 2 \pi \text{ tubuleLength tubuleRad}$$

```
totCurvedArea[initialSheetRad_, thickness_,
  tubuleRad_, holeRad_, tubuleLength_, nHoles_] =
 CurvatureUnholey[initialSheetRad, thickness] + δCurvatureTubule[tubuleRad,
   tubuleLength] + nHoles * δCurvatureHole[holeRad, thickness]
```

$$\text{nHoles } \pi^2 \text{ thickness} \left(\text{holeRad} - \frac{2 \text{ thickness}}{3 \pi}\right) + \pi^2 \text{ thickness} \left(\text{initialSheetRad} + \frac{2 \text{ thickness}}{3 \pi}\right) + 2 \pi \text{ tubuleLength tubuleRad}$$

Case of Constant Surface Area

We have to go about this a little differently to keep surface area constant. We want to let the initial sheet radius float, such that any changes due to adding a tubule, or hole do not change the total surface area. We need the sheet disk radius as a function of everything else.

```
Solve[totSurfaceArea[sheetRad, thickness, tubuleRad,
   holeRad, tubuleLength, nHoles] == constantSurfaceArea, sheetRad]
```

$$\left\{\left\{\text{sheetRad}\to \frac{1}{4\pi}\left(-\pi^2\,\text{thickness}-\sqrt{\frac{\pi}{3}}\sqrt{\left(24\,\text{constantSurfaceArea}+48\,\text{holeRad}^2\,\text{nHoles}\,\pi-24\,\text{holeRad}\,\text{nHoles}\,\pi^2\,\text{thickness}+16\,\text{nHoles}\,\pi\,\text{thickness}^2+\frac{\left(-16\,\pi^2+3\,\pi^4\right)\text{thickness}^2}{\pi}-48\,\pi\,\text{tubuleLength}\,\text{tubuleRad}\right)}\right)\right\},\right.$$

$$\left.\left\{\text{sheetRad}\to \frac{1}{4\pi}\left(-\pi^2\,\text{thickness}+\sqrt{\frac{\pi}{3}}\sqrt{\left(24\,\text{constantSurfaceArea}+48\,\text{holeRad}^2\,\text{nHoles}\,\pi-24\,\text{holeRad}\,\text{nHoles}\,\pi^2\,\text{thickness}+16\,\text{nHoles}\,\pi\,\text{thickness}^2+\frac{\left(-16\,\pi^2+3\,\pi^4\right)\text{thickness}^2}{\pi}-48\,\pi\,\text{tubuleLength}\,\text{tubuleRad}\right)}\right)\right\}\right\}$$

Take the one which has positive radii, i.e. sheetRadiusPlus.

```
sheetRadiusPlus[thickness_, tubuleRad_,
  holeRad_, tubuleLength_, nHoles_] = FullSimplify[
```

$$\frac{1}{4\pi}\left(-\pi^2\,\text{thickness}+\sqrt{\frac{\pi}{3}}\sqrt{\left(24\,\text{constantSurfaceArea}+48\,\text{holeRad}^2\,\text{nHoles}\,\pi-24\,\text{holeRad}\,\text{nHoles}\,\pi^2\,\text{thickness}+16\,\text{nHoles}\,\pi\,\text{thickness}^2+\frac{\left(-16\,\pi^2+3\,\pi^4\right)\text{thickness}^2}{\pi}-48\,\pi\,\text{tubuleLength}\,\text{tubuleRad}\right)}\right)]$$

$$-\frac{\pi\,\text{thickness}}{4}+\frac{1}{4}\sqrt{\left(16\,\text{holeRad}^2\,\text{nHoles}+\frac{8\,\text{constantSurfaceArea}}{\pi}-8\,\text{holeRad}\,\text{nHoles}\,\pi\,\text{thickness}+\frac{16}{3}\left(-1+\text{nHoles}\right)\text{thickness}^2+\pi^2\,\text{thickness}^2-16\,\text{tubuleLength}\,\text{tubuleRad}\right)}$$

```
totVolume[thickness_, tubuleRad_, holeRad_, tubuleLength_, nHoles_] =
 Volunholey[sheetRadiusPlus[thickness,
    tubuleRad, holeRad, tubuleLength, nHoles], thickness] +
  δVtub[tubuleRad, tubuleLength] + nHoles * δVhole[holeRad, thickness]
```

$$\text{initialSheetRad}^2\,\pi\,\text{thickness} + \frac{1}{4}\pi^2\,\text{thickness}^2\left(\text{initialSheetRad} + \frac{2\,\text{thickness}}{3\,\pi}\right) + \text{nHoles}\left(-\text{holeRad}^2\,\pi\,\text{thickness} + \frac{1}{4}\pi^2\,\text{thickness}^2\left(\text{holeRad} - \frac{2\,\text{thickness}}{3\,\pi}\right)\right) + \pi\,\text{tubuleLength}\,\text{tubuleRad}^2$$

$$\text{nHoles}\left(-\text{holeRad}^2\,\pi\,\text{thickness} + \frac{1}{4}\pi^2\,\text{thickness}^2\left(\text{holeRad} - \frac{2\,\text{thickness}}{3\,\pi}\right)\right) + \pi\,\text{tubuleLength}\,\text{tubuleRad}^2 + \pi\,\text{thickness}\left(-\frac{\pi\,\text{thickness}}{4} + \frac{1}{4}\sqrt{\left(16\,\text{holeRad}^2\,\text{nHoles} + \frac{8\,\text{constantSurfaceArea}}{\pi} - 8\,\text{holeRad}\,\text{nHoles}\,\pi\,\text{thickness} + \frac{16}{3}\left(-1 + \text{nHoles}\right)\text{thickness}^2 + \pi^2\,\text{thickness}^2 - 16\,\text{tubuleLength}\,\text{tubuleRad}\right)}\right)^2 + \frac{1}{4}\pi^2\,\text{thickness}^2\left(\frac{2\,\text{thickness}}{3\,\pi} - \frac{\pi\,\text{thickness}}{4} + \frac{1}{4}\sqrt{\left(16\,\text{holeRad}^2\,\text{nHoles} + \frac{8\,\text{constantSurfaceArea}}{\pi} - 8\,\text{holeRad}\,\text{nHoles}\,\pi\,\text{thickness} + \frac{16}{3}\left(-1 + \text{nHoles}\right)\text{thickness}^2 + \pi^2\,\text{thickness}^2 - 16\,\text{tubuleLength}\,\text{tubuleRad}\right)}\right)$$

$$\text{initialSheetRad}^2\,\pi\,\text{thickness} + \frac{1}{4}\pi^2\,\text{thickness}^2\left(\text{initialSheetRad} + \frac{2\,\text{thickness}}{3\,\pi}\right) + \text{nHoles}\left(-\text{holeRad}^2\,\pi\,\text{thickness} + \frac{1}{4}\pi^2\,\text{thickness}^2\left(\text{holeRad} - \frac{2\,\text{thickness}}{3\,\pi}\right)\right) + \pi\,\text{tubuleLength}\,\text{tubuleRad}^2$$

```
totSurfaceArea[thickness_, tubuleRad_, holeRad_, tubuleLength_, nHoles_] =
 SAunholey[sheetRadiusPlus[thickness, tubuleRad, holeRad, tubuleLength, nHoles],
   thickness] + δSAtub[tubuleRad, tubuleLength] +
  nHoles * δSAhole[holeRad, thickness]
```

$$2\,\text{initialSheetRad}^2\,\pi + \pi^2\,\text{thickness}\left(\text{initialSheetRad} + \frac{2\,\text{thickness}}{3\,\pi}\right) + \text{nHoles}\left(-2\,\text{holeRad}^2\,\pi + \pi^2\,\text{thickness}\left(\text{holeRad} - \frac{2\,\text{thickness}}{3\,\pi}\right)\right) + 2\,\pi\,\text{tubuleLength}\,\text{tubuleRad}$$

$$\text{nHoles}\left(-2\,\text{holeRad}^2\,\pi + \pi^2\,\text{thickness}\left(\text{holeRad} - \frac{2\,\text{thickness}}{3\,\pi}\right)\right) + 2\,\pi\,\text{tubuleLength}\,\text{tubuleRad} + 2\,\pi\left(-\frac{\pi\,\text{thickness}}{4} + \frac{1}{4}\sqrt{\left(16\,\text{holeRad}^2\,\text{nHoles} + \frac{8\,\text{constantSurfaceArea}}{\pi} - 8\,\text{holeRad}\,\text{nHoles}\,\pi\,\text{thickness} + \frac{16}{3}\left(-1 + \text{nHoles}\right)\text{thickness}^2 + \pi^2\,\text{thickness}^2 - 16\,\text{tubuleLength}\,\text{tubuleRad}\right)}\right)^2 + \pi^2\,\text{thickness}\left(\frac{2\,\text{thickness}}{3\,\pi} - \frac{\pi\,\text{thickness}}{4} + \frac{1}{4}\sqrt{\left(16\,\text{holeRad}^2\,\text{nHoles} + \frac{8\,\text{constantSurfaceArea}}{\pi} - 8\,\text{holeRad}\,\text{nHoles}\,\pi\,\text{thickness} + \frac{16}{3}\left(-1 + \text{nHoles}\right)\text{thickness}^2 + \pi^2\,\text{thickness}^2 - 16\,\text{tubuleLength}\,\text{tubuleRad}\right)}\right)$$

$$2\,\text{initialSheetRad}^2\,\pi + \pi^2\,\text{thickness}\left(\text{initialSheetRad} + \frac{2\,\text{thickness}}{3\,\pi}\right) + \text{nHoles}\left(-2\,\text{holeRad}^2\,\pi + \pi^2\,\text{thickness}\left(\text{holeRad} - \frac{2\,\text{thickness}}{3\,\pi}\right)\right) + 2\,\pi\,\text{tubuleLength}\,\text{tubuleRad}$$

```
totCurvedArea[thickness_, tubuleRad_, holeRad_, tubuleLength_, nHoles_] =
 CurvatureUnholey[sheetRadiusPlus[thickness, tubuleRad,
    holeRad, tubuleLength, nHoles], thickness] + δCurvatureTubule[
   tubuleRad, tubuleLength] + nHoles * δCurvatureHole[holeRad, thickness]
```

$$\text{nHoles}\,\pi^2\,\text{thickness}\left(\text{holeRad}-\frac{2\,\text{thickness}}{3\,\pi}\right)+\pi^2\,\text{thickness}\left(\text{initialSheetRad}+\frac{2\,\text{thickness}}{3\,\pi}\right)+\delta\text{SAtub}[\text{tubuleRad},\ \text{tubuleLength}]$$

$$\text{nHoles}\,\pi^2\,\text{thickness}\left(\text{holeRad}-\frac{2\,\text{thickness}}{3\,\pi}\right)+2\,\pi\,\text{tubuleLength}\,\text{tubuleRad}+\pi^2\,\text{thickness}\left(\frac{2\,\text{thickness}}{3\,\pi}-\frac{\pi\,\text{thickness}}{4}+\frac{1}{4}\sqrt{\left(16\,\text{holeRad}^2\,\text{nHoles}+\frac{8\,\text{constantSurfaceArea}}{\pi}-8\,\text{holeRad}\,\text{nHoles}\,\pi\,\text{thickness}+\frac{16}{3}\left(-1+\text{nHoles}\right)\text{thickness}^2+\pi^2\,\text{thickness}^2-16\,\text{tubuleLength}\,\text{tubuleRad}\right)}\right)$$

$$\text{nHoles}\,\pi^2\,\text{thickness}\left(\text{holeRad}-\frac{2\,\text{thickness}}{3\,\pi}\right)+\pi^2\,\text{thickness}\left(\text{initialSheetRad}+\frac{2\,\text{thickness}}{3\,\pi}\right)+2\,\pi\,\text{tubuleLength}\,\text{tubuleRad}$$

small plotting things

```
sheetRadiusColorMinimum = 2427; sheetRadiusColorMaximum = 2515;
```

Fixing surface area at that of an unholey sheet with radius 2.5 μm, thickness of 30 nm

```
sa30nm = SAunholey[2.5 * 10^3, 30] // N
vol30nm =
 totVolume[30, 50 , 50 + (0.5 * 30), 0, 0] /. {constantSurfaceArea → sa30nm} // N
```

4.0012×10^{7}

5.94614×10^{8}

Recall: bigR is the radius of the flat part that is cut out of the sheet, and little r is half the thickness of the sheet. Function signature is [thickness_, tubuleRad_, holeRad_, tubuleLength_, nHoles_]

In order to specify the inner radius of the hole (additionally ensuring there is actually a hole), we need to change the holeRad input to be (holeRad + 0.5 * thickness). This is essentially a change of variables to bump the inner radius of the hole from (holeRad - 0.5*thickness) to the specified inner radius (one could call it holeRad’ or so).

```
dp = DensityPlot[1/vol30nm totVolume[30, 50 , 50 + (0.5 * 30), tlength, nholes] /.
     {constantSurfaceArea → sa30nm},
    {tlength, 0, 5000}, {nholes, 0, 50}, PlotLegends → Automatic,
    FrameLabel → {"Length of Tubule [nm]", "Number of r=50 nm Holes"},
    PlotLabel → "Normalized Enclosed Volume", ColorFunction → "SunsetColors"];
cp = ContourPlot[1/vol30nm totVolume[30, 50 , 50 + (0.5 * 30), tlength, nholes] /.
     {constantSurfaceArea → sa30nm},
    {tlength, 0, 5000}, {nholes, 0, 50}, PlotLegends → Automatic,
    FrameLabel → {"Length of Tubule [nm]", "Number of r=50 nm Holes"},
    PlotLabel → "Normalized Enclosed Volume", ColorFunction → "SunsetColors",
    ContourShading → None, ContourStyle → White, PlotPoints → 200];
cpl = ContourPlot[1/vol30nm totVolume[30, 50 , 50 + (0.5 * 30), tlength, nholes] /.
    {constantSurfaceArea → sa30nm},
   {tlength, 0, 5000}, {nholes, 0, 50}, PlotLegends → Automatic,
   FrameLabel → {"Length of Tubule [nm]", "Number of r=50 nm Holes"},
   PlotLabel → "Normalized Enclosed Volume", ColorFunction → "SunsetColors",
   ContourShading → None, ContourLabels → True]
Show[
 {dp,
  cp}]
```

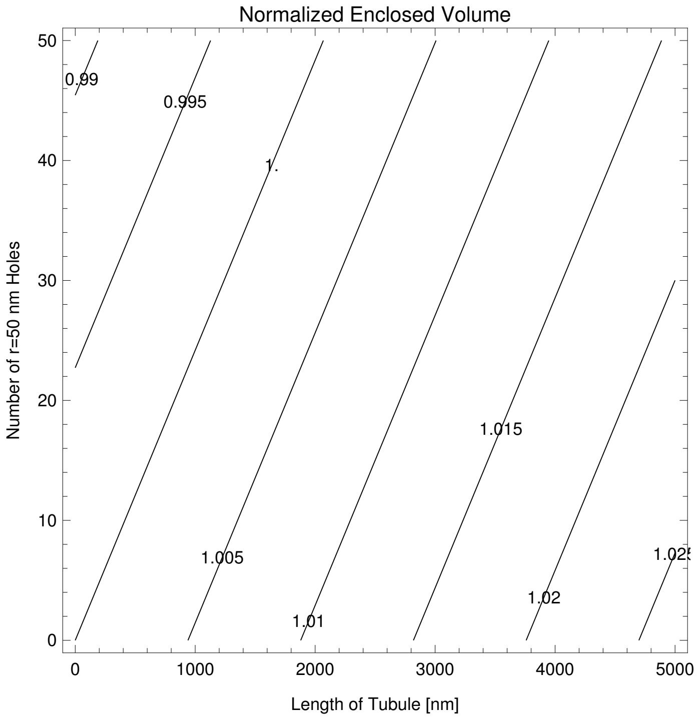

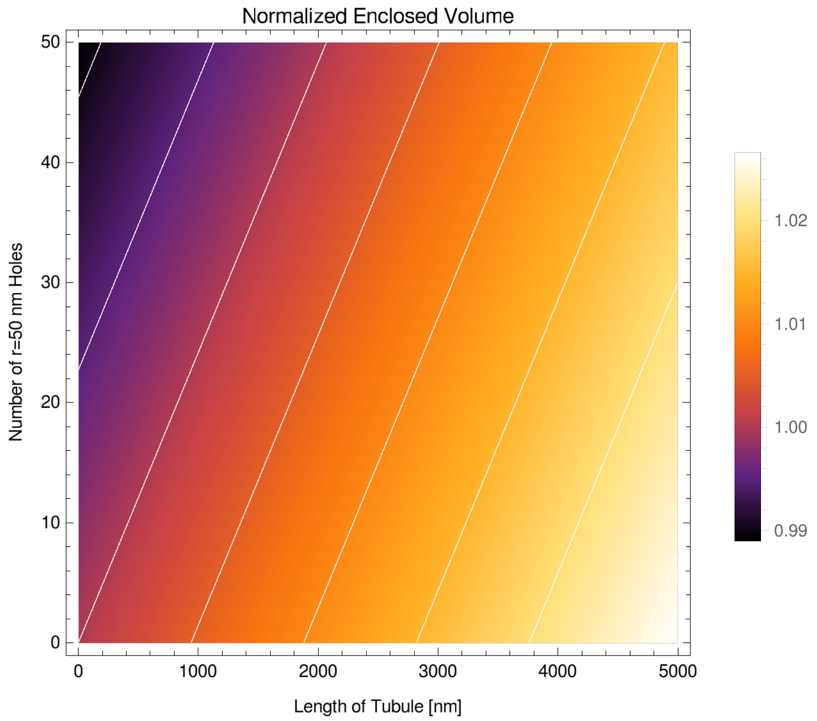


```
dp = DensityPlot[1/sa30nm (totCurvedArea[30, 50, 50 + (0.5 * 30), tlength, nholes] /.
      {constantSurfaceArea → sa30nm}),
   {tlength, 0, 5000}, {nholes, 0, 50}, PlotLegends → Automatic,
   FrameLabel → {"Length of Tubule [nm]", "Number of r=50 nm Holes"},
   PlotLabel → "Curved Fraction of Surface Area",
   ColorFunction → "SunsetColors", PlotPoints → 200];
cpl = ContourPlot[1/sa30nm (totCurvedArea[30, 50, 50 + (0.5 * 30), tlength, nholes] /.
     {constantSurfaceArea → sa30nm}),
  {tlength, 0, 5000}, {nholes, 0, 50}, PlotLegends → Automatic,
  FrameLabel → {"Length of Tubule [nm]", "Number of r=50 nm Holes"},
  PlotLabel → "Curved Fraction of Surface Area",
  ColorFunction → "SunsetColors", ContourShading → None, ContourLabels → True]
cp = ContourPlot[1/sa30nm (totCurvedArea[30, 50, 50 + (0.5 * 30), tlength, nholes] /.
      {constantSurfaceArea → sa30nm}),
   {tlength, 0, 5000}, {nholes, 0, 50}, PlotLegends → Automatic,
   FrameLabel → {"Length of Tubule [nm]", "Number of r=50 nm Holes"},
   PlotLabel → "Curved Fraction of Surface Area", ColorFunction → "SunsetColors",
   ContourShading → None, ContourStyle → White, PlotPoints → 200];
Show[
 {dp,
  cp}]
```

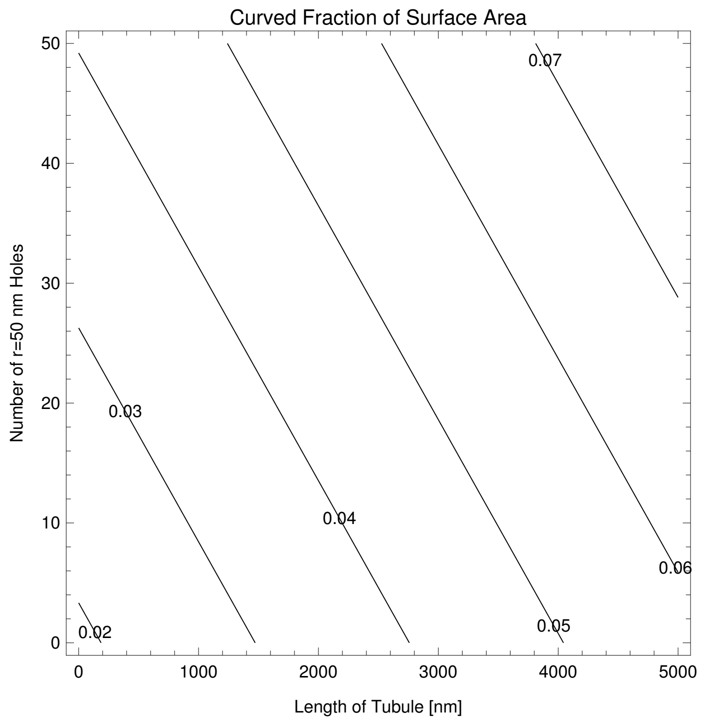
Curved Fraction of Surface Area
Number of r=50 nm Holes
Length of Tubule [nm]
0.02
0.03
0.04
0.05
0.06
0.07
0
10
20
30
40
50
0
1000
2000
3000
4000
5000

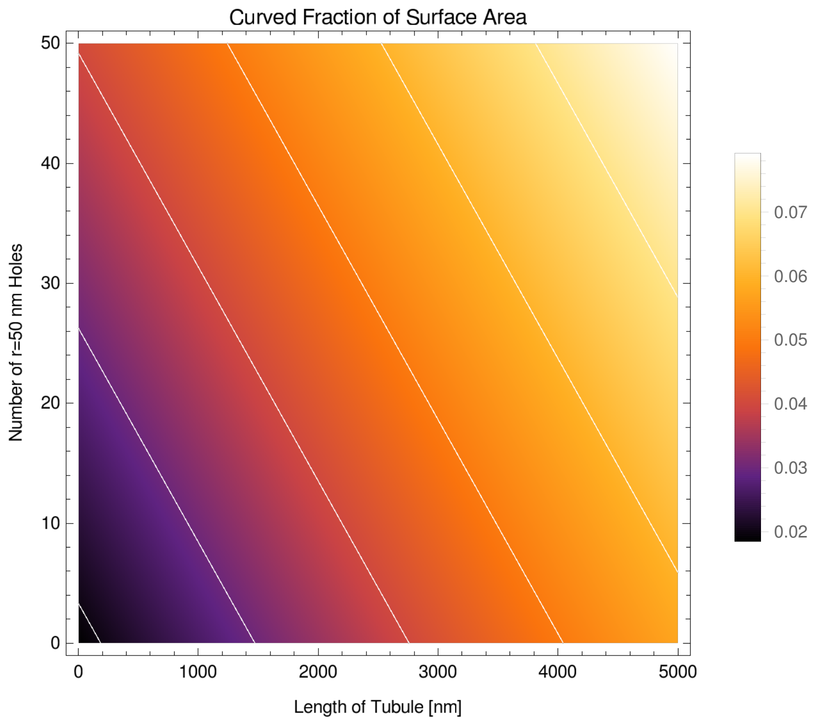
Curved Fraction of Surface Area
Number of r=50 nm Holes
Length of Tubule [nm]
0
10
20
30
40
50
0
1000
2000
3000
4000
5000
0.07
0.06
0.05
0.04
0.03
0.02

```
dp = DensityPlot[(sheetRadiusPlus[30, 50, 50 + (0.5 * 30), tlength, nholes] /.
      {constantSurfaceArea → sa30nm}),
    {tlength, 0, 5000}, {nholes, 0, 50}, PlotLegends → Automatic,
    FrameLabel → {"Length of Tubule [nm]", "Number of r=50 nm Holes"},
    PlotLabel → "Sheet Radius", ColorFunction → (ColorData["SunsetColors"][
        Rescale[#, {sheetRadiusColorMinimum, sheetRadiusColorMaximum}]] &),
    ColorFunctionScaling → False, PlotPoints → 200];
cp = ContourPlot[(sheetRadiusPlus[30, 50, 50 + (0.5 * 30), tlength, nholes] /.
      {constantSurfaceArea → sa30nm}),
    {tlength, 0, 5000}, {nholes, 0, 50}, PlotLegends → Automatic,
    FrameLabel → {"Length of Tubule [nm]", "Number of r=50 nm Holes"},
    PlotLabel → "Sheet Radius", ColorFunction → "SunsetColors",
    ContourShading → None, ContourStyle → White, PlotPoints → 200];
cpl = ContourPlot[(sheetRadiusPlus[30, 50, 50 + (0.5 * 30), tlength, nholes] /.
     {constantSurfaceArea → sa30nm}),
   {tlength, 0, 5000}, {nholes, 0, 50}, PlotLegends → Automatic,
   FrameLabel → {"Length of Tubule [nm]", "Number of r=50 nm Holes"},
   PlotLabel → "Sheet Radius", ColorFunction → "SunsetColors",
   ContourShading → None, ContourLabels → True]
Show[
 dp,
 cp]
```

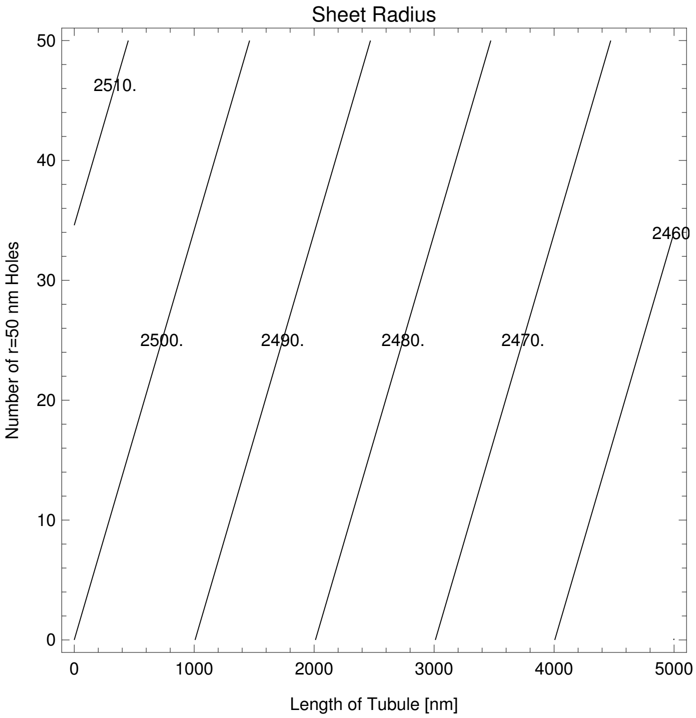

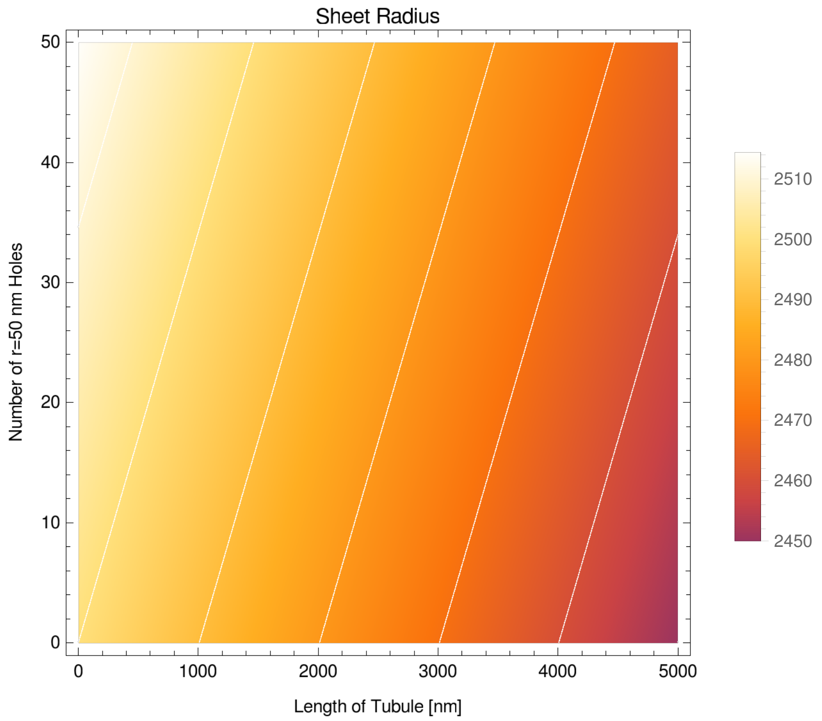

Initial sheet radius of 2.5 μm, thickness of 50 nm

```
sa50nm = SAunholey[2.5 * 10^3, 50] // N
vol50nm =
 totVolume[50, 50, 50 + (0.5 * 50), 0, 0] /. {constantSurfaceArea → sa50nm} // N
```

4.05088×10^{7}

9.97234×10^{8}

```
dp = DensityPlot[ 1/vol50nm totVolume[50, 50, 50 + (0.5 * 50), tlength, nholes] /.
     {constantSurfaceArea → sa50nm},
    {tlength, 0, 5000}, {nholes, 0, 50}, PlotLegends → Automatic,
    FrameLabel → {"Length of Tubule [nm]", "Number of r=50 nm Holes"},
    PlotLabel → "Normalized Enclosed Volume",
    ColorFunction → "SunsetColors", PlotPoints → 200];
cp = ContourPlot[ 1/vol50nm totVolume[50, 50, 50 + (0.5 * 50), tlength, nholes] /.
     {constantSurfaceArea → sa50nm},
    {tlength, 0, 5000}, {nholes, 0, 50}, PlotLegends → Automatic,
    FrameLabel → {"Length of Tubule [nm]", "Number of r=50 nm Holes"},
    PlotLabel → "Normalized Enclosed Volume", ColorFunction → "SunsetColors",
    ContourShading → None, ContourStyle → White, PlotPoints → 200];
cpl = ContourPlot[ 1/vol50nm totVolume[50, 50, 50 + (0.5 * 50), tlength, nholes] /.
    {constantSurfaceArea → sa50nm},
   {tlength, 0, 5000}, {nholes, 0, 50}, PlotLegends → Automatic,
   FrameLabel → {"Length of Tubule [nm]", "Number of r=50 nm Holes"},
   PlotLabel → "Normalized Enclosed Volume", ColorFunction → "SunsetColors",
   ContourShading → None, ContourLabels → True]
Show[
 dp,
 cp]
```

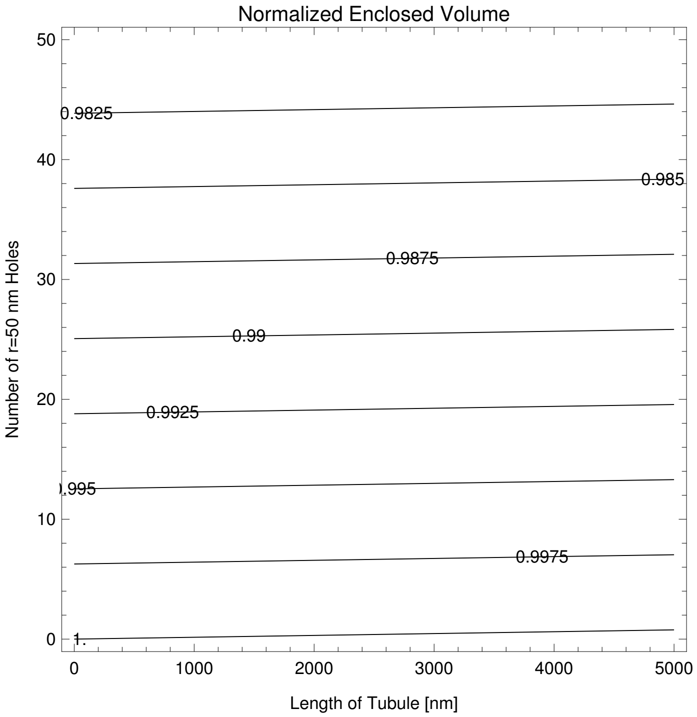

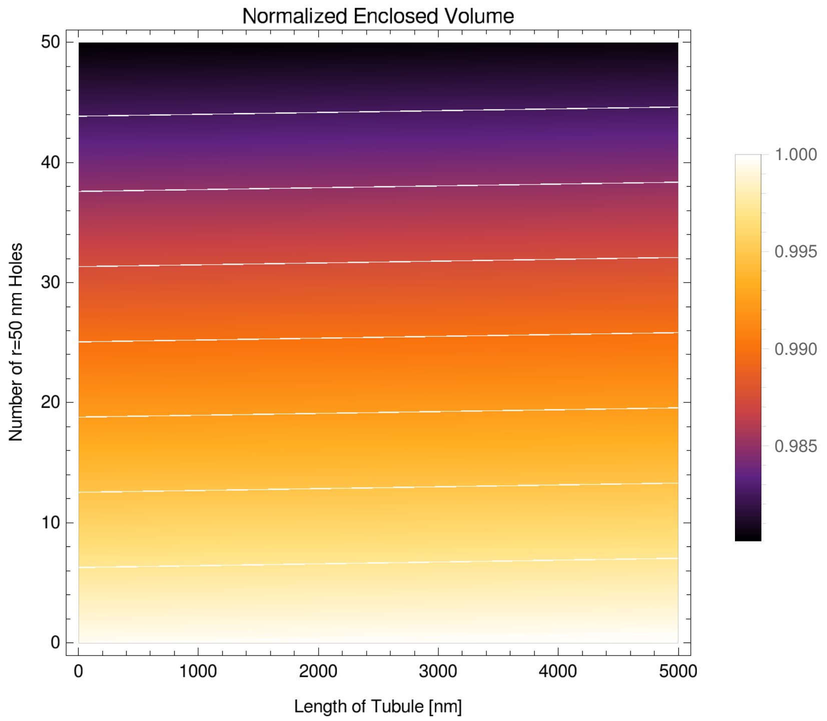


```
dp = DensityPlot[1/sa50nm (totCurvedArea[50, 50, 50 + (0.5 * 50), tlength, nholes] /.
      {constantSurfaceArea → sa50nm}),
    {tlength, 0, 5000}, {nholes, 0, 50}, PlotLegends → Automatic,
    FrameLabel → {"Length of Tubule [nm]", "Number of r=50 nm Holes"},
    PlotLabel → "Curved Fraction of Surface Area",
    ColorFunction → "SunsetColors", PlotPoints → 200];
cp = ContourPlot[1/sa50nm (totCurvedArea[50, 50, 50 + (0.5 * 50), tlength, nholes] /.
      {constantSurfaceArea → sa50nm}),
    {tlength, 0, 5000}, {nholes, 0, 50}, PlotLegends → Automatic,
    FrameLabel → {"Length of Tubule [nm]", "Number of r=50 nm Holes"},
    PlotLabel → "Curved Fraction of Surface Area", ColorFunction → "SunsetColors",
    ContourShading → None, ContourStyle → White, PlotPoints → 200];
cpl = ContourPlot[1/sa50nm (totCurvedArea[50, 50, 50 + (0.5 * 50), tlength, nholes] /.
      {constantSurfaceArea → sa50nm}),
   {tlength, 0, 5000}, {nholes, 0, 50}, PlotLegends → Automatic,
   FrameLabel → {"Length of Tubule [nm]", "Number of r=50 nm Holes"},
   PlotLabel → "Curved Fraction of Surface Area",
   ColorFunction → "SunsetColors", ContourShading → None, ContourLabels → True]
Show[dp, cp]
```

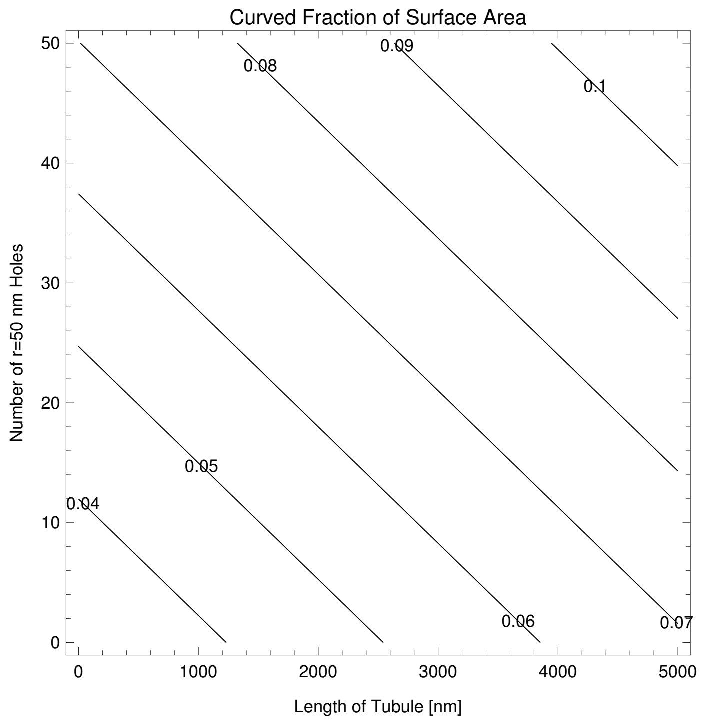
Curved Fraction of Surface Area
Number of r=50 nm Holes
Length of Tubule [nm]
0.04
0.05
0.06
0.07
0.08
0.09
0.1
0
10
20
30
40
50
0
1000
2000
3000
4000
5000

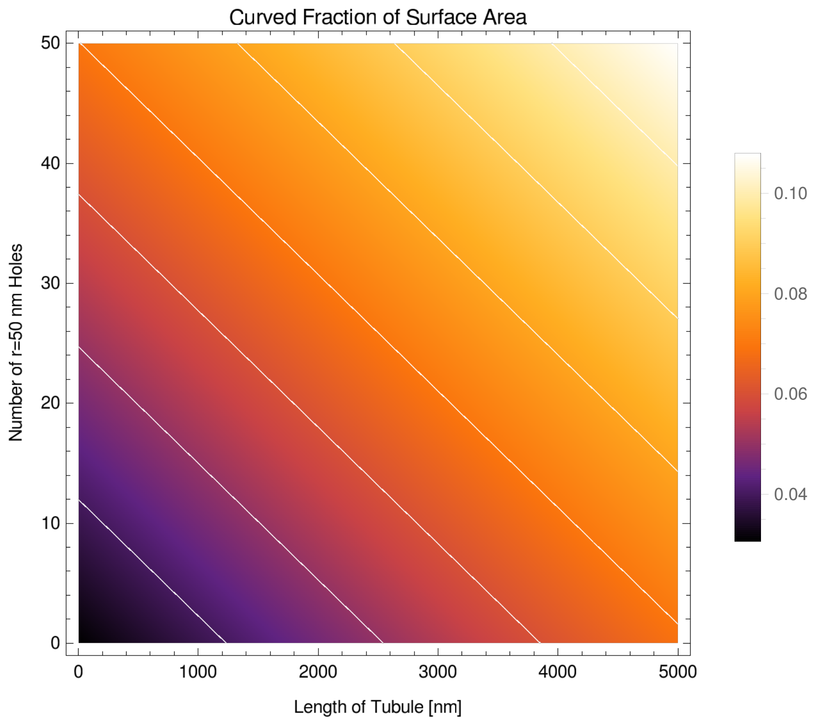
Curved Fraction of Surface Area
Number of r=50 nm Holes
Length of Tubule [nm]
0
10
20
30
40
50
0
1000
2000
3000
4000
5000
0.10
0.08
0.06
0.04

```
cp = ContourPlot[1/sa50nm (totCurvedArea[50, 50, 50 + (0.5 * 50), tlength, nholes] /.
       {constantSurfaceArea → sa50nm}) == 0.07,
   {tlength, 0, 5000}, {nholes, 0, 50}, PlotLegends → Automatic,
   FrameLabel → {"Length of Tubule [nm]", "Number of r=50 nm Holes"},
   PlotLabel → "Curved Fraction of Surface Area",
   ColorFunction → "SunsetColors", ContourShading → None, ContourStyle → Black];
sp = ListPlot[{{4855, 3}, {2481, 26}, {218, 48}}, PlotMarkers → {■, Large}];
Show[cp, sp]
```

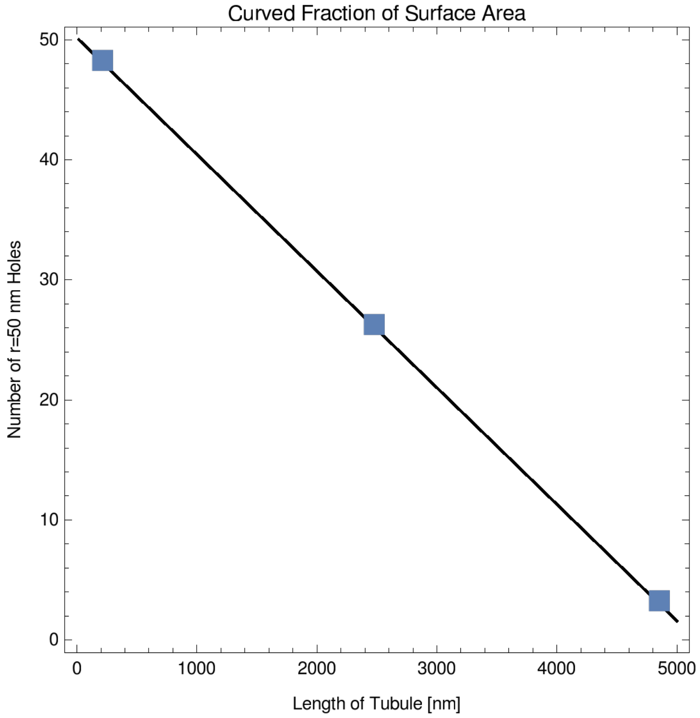

```
dp = DensityPlot[(sheetRadiusPlus[50, 50, 50 + (0.5 * 50), tlength, nholes] /.
      {constantSurfaceArea → sa50nm}),
    {tlength, 0, 5000}, {nholes, 0, 50}, PlotLegends → Automatic,
    FrameLabel → {"Length of Tubule [nm]", "Number of r=50 nm Holes"},
    PlotLabel → "Sheet Radius", ColorFunction →
     (ColorData["SunsetColors"][Rescale[#, {sheetRadiusColorMinimum,
          sheetRadiusColorMaximum}]] &), ColorFunctionScaling → False];
cp = ContourPlot[(sheetRadiusPlus[50, 50, 50 + (0.5 * 50), tlength, nholes] /.
      {constantSurfaceArea → sa50nm}),
    {tlength, 0, 5000}, {nholes, 0, 50}, PlotLegends → Automatic,
    FrameLabel → {"Length of Tubule [nm]", "Number of r=50 nm Holes"},
    PlotLabel → "Sheet Radius", ColorFunction → "SunsetColors",
    ContourShading → None, ContourStyle → White, PlotPoints → 200];
cpl = ContourPlot[(sheetRadiusPlus[50, 50, 50 + (0.5 * 50), tlength, nholes] /.
     {constantSurfaceArea → sa50nm}),
   {tlength, 0, 5000}, {nholes, 0, 50}, PlotLegends → Automatic,
   FrameLabel → {"Length of Tubule [nm]", "Number of r=50 nm Holes"},
   PlotLabel → "Sheet Radius", ColorFunction → "SunsetColors",
   ContourShading → None, ContourLabels → True]
Show[
 dp,
 cp]
```

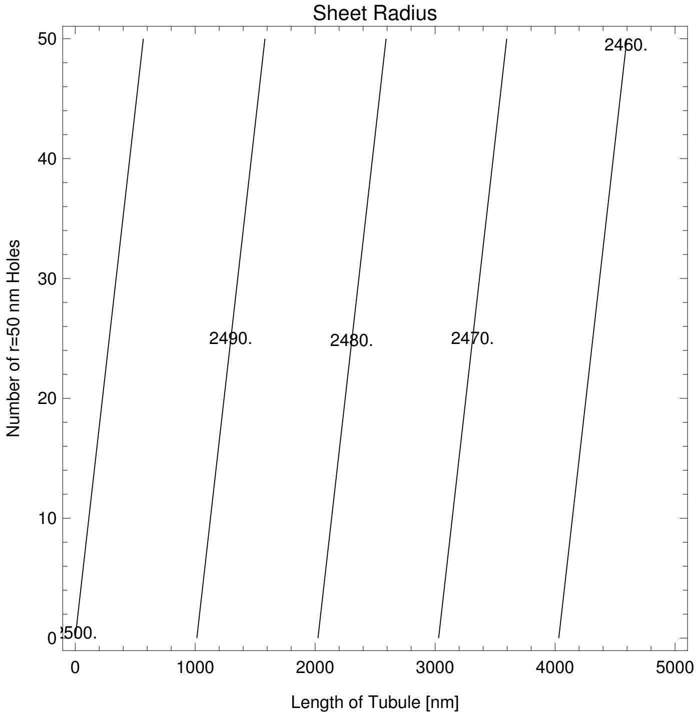

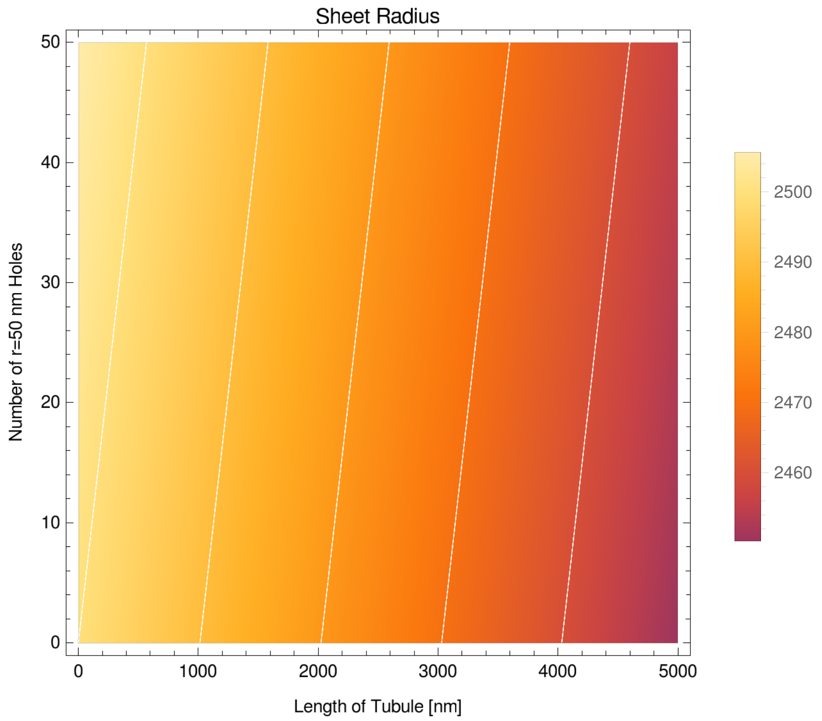

Initial sheet radius of 2.5 μm, thickness of 100 nm

```
sa100nm = SAunholey[2.5 * 10^3, 100] // N
vol100nm =
 totVolume[100, 50, 50 + (0.5 * 100), 0, 0] /. {constantSurfaceArea → sa100nm} // N
```

4.17583×10^{7}

2.0257×10^{9}

```
dp = DensityPlot[1/vol100nm totVolume[100, 50, 50 + (0.5 * 100), tlength, nholes] /.
     {constantSurfaceArea → sa100nm},
    {tlength, 0, 5000}, {nholes, 0, 50}, PlotLegends → Automatic,
    FrameLabel → {"Length of Tubule [nm]", "Number of r=50 nm Holes"},
    PlotLabel → "Normalized Enclosed Volume",
    ColorFunction → "SunsetColors", PlotPoints → 200];
cp = ContourPlot[1/vol100nm totVolume[100, 50, 50 + (0.5 * 100), tlength, nholes] /.
     {constantSurfaceArea → sa100nm},
    {tlength, 0, 5000}, {nholes, 0, 50}, PlotLegends → Automatic,
    FrameLabel → {"Length of Tubule [nm]", "Number of r=50 nm Holes"},
    PlotLabel → "Normalized Enclosed Volume", ColorFunction → "SunsetColors",
    ContourShading → None, ContourStyle → White, PlotPoints → 200];
cpl = ContourPlot[1/vol100nm totVolume[100, 50, 50 + (0.5 * 100), tlength, nholes] /.
    {constantSurfaceArea → sa100nm},
   {tlength, 0, 5000}, {nholes, 0, 50}, PlotLegends → Automatic,
   FrameLabel → {"Length of Tubule [nm]", "Number of r=50 nm Holes"},
   PlotLabel → "Normalized Enclosed Volume", ColorFunction → "SunsetColors",
   ContourShading → None, ContourLabels → True]
Show[
 dp,
 cp]
```

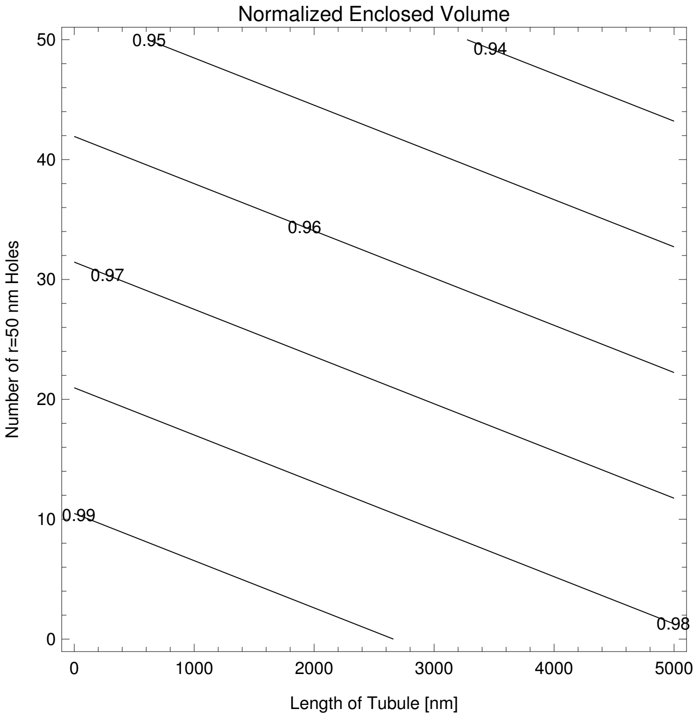

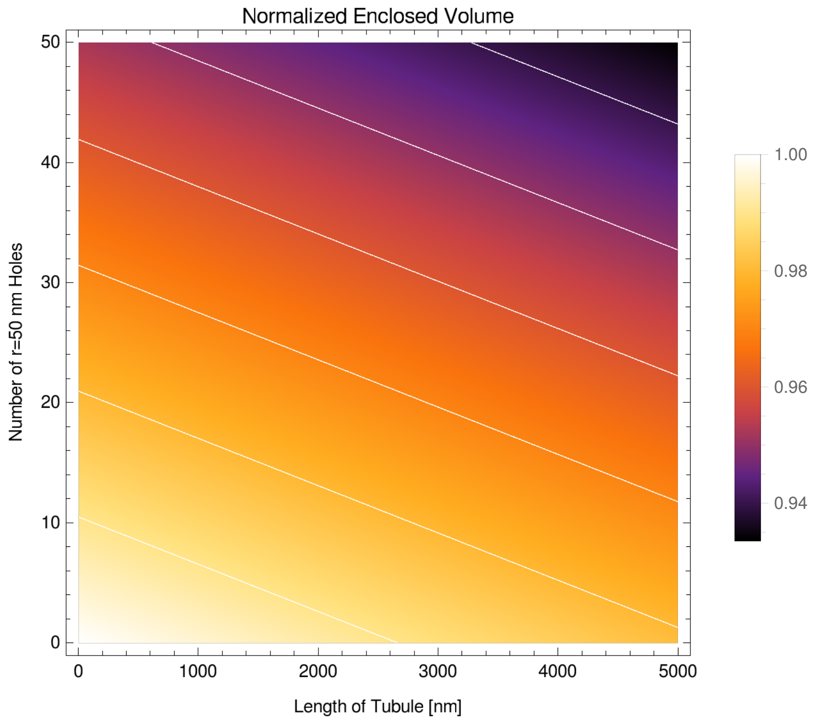


```
dp =
  DensityPlot[1/sa100nm (totCurvedArea[100, 50, 50 + (0.5 * 100), tlength, nholes] /.
      {constantSurfaceArea → sa100nm}),
   {tlength, 0, 5000}, {nholes, 0, 50}, PlotLegends → Automatic,
   FrameLabel → {"Length of Tubule [nm]", "Number of r=50 nm Holes"},
   PlotLabel → "Curved Fraction of Surface Area",
   ColorFunction → "SunsetColors", PlotPoints → 200];
cp = ContourPlot[1/sa100nm (totCurvedArea[100, 50, 50 + (0.5 * 100),
       tlength, nholes] /. {constantSurfaceArea → sa100nm}),
   {tlength, 0, 5000}, {nholes, 0, 50}, PlotLegends → Automatic,
   FrameLabel → {"Length of Tubule [nm]", "Number of r=50 nm Holes"},
   PlotLabel → "Curved Fraction of Surface Area", ColorFunction → "SunsetColors",
   ContourShading → None, ContourStyle → White, PlotPoints → 200];
cpl = ContourPlot[1/sa100nm (totCurvedArea[100, 50, 50 + (0.5 * 100),
      tlength, nholes] /. {constantSurfaceArea → sa100nm}),
  {tlength, 0, 5000}, {nholes, 0, 50}, PlotLegends → Automatic,
  FrameLabel → {"Length of Tubule [nm]", "Number of r=50 nm Holes"},
  PlotLabel → "Curved Fraction of Surface Area",
  ColorFunction → "SunsetColors", ContourShading → None, ContourLabels → True]
Show[
 dp,
 cp]
```

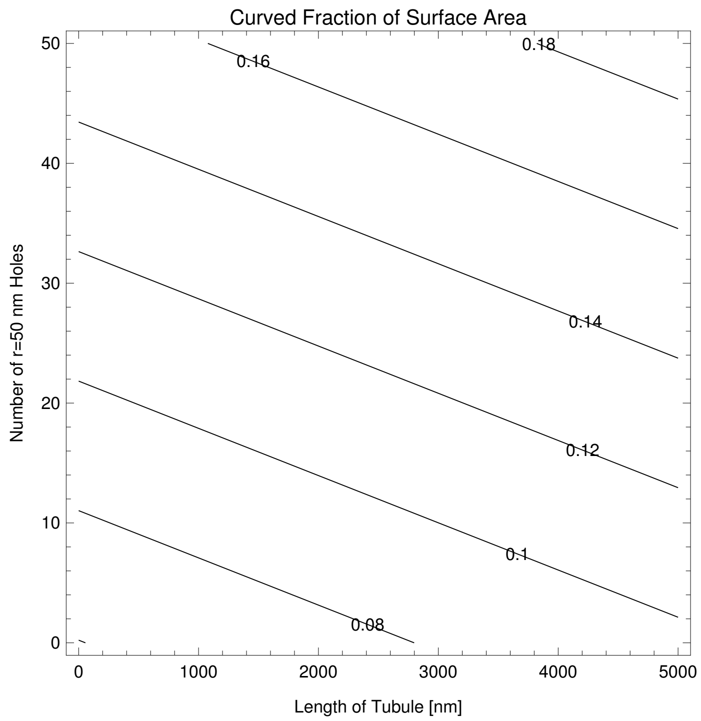
Curved Fraction of Surface Area
0.16
0.18
0.14
0.12
0.1
0.08
Number of r=50 nm Holes
Length of Tubule [nm]
0
10
20
30
40
50
1000
2000
3000
4000
5000

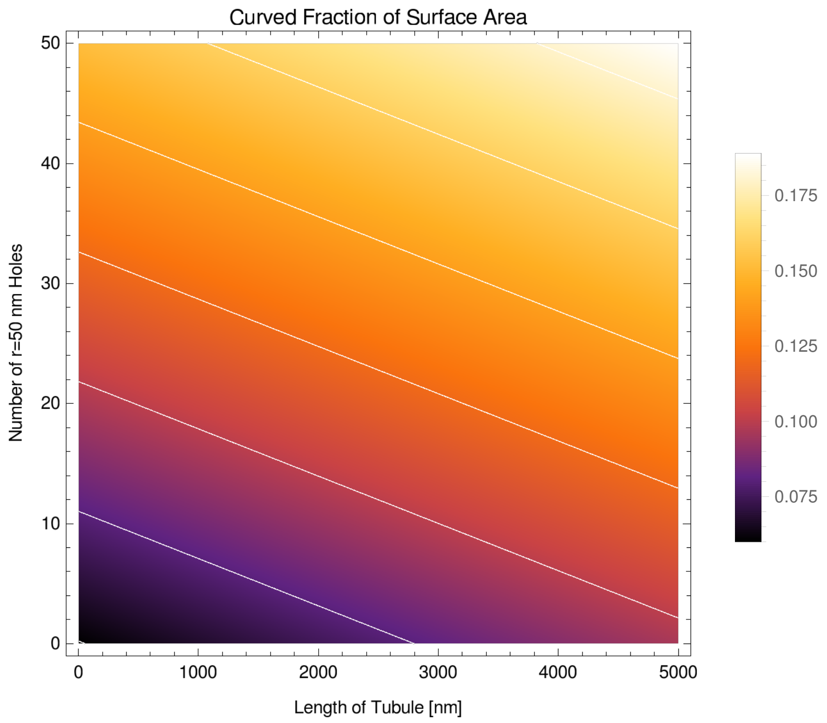
Curved Fraction of Surface Area
Number of r=50 nm Holes
Length of Tubule [nm]
0
10
20
30
40
50
1000
2000
3000
4000
5000
0.175
0.150
0.125
0.100
0.075

```
dp = DensityPlot[(sheetRadiusPlus[100, 50, 50 + (0.5 * 100), tlength, nholes] /.
      {constantSurfaceArea → sa100nm}),
    {tlength, 0, 5000}, {nholes, 0, 50}, PlotLegends → Automatic,
    FrameLabel → {"Length of Tubule [nm]", "Number of r=50 nm Holes"},
    PlotLabel → "Sheet Radius", ColorFunction → (ColorData["SunsetColors"][
        Rescale[#, {sheetRadiusColorMinimum, sheetRadiusColorMaximum}]] &),
    ColorFunctionScaling → False, PlotPoints → 200];
cp = ContourPlot[(sheetRadiusPlus[100, 50, 50 + (0.5 * 100), tlength, nholes] /.
      {constantSurfaceArea → sa100nm}),
    {tlength, 0, 5000}, {nholes, 0, 50}, PlotLegends → Automatic,
    FrameLabel → {"Length of Tubule [nm]", "Number of r=50 nm Holes"},
    PlotLabel → "Sheet Radius", ColorFunction → "SunsetColors",
    ContourShading → None, ContourStyle → White, PlotPoints → 200];
cpl = ContourPlot[(sheetRadiusPlus[100, 50, 50 + (0.5 * 100), tlength, nholes] /.
     {constantSurfaceArea → sa100nm}),
   {tlength, 0, 5000}, {nholes, 0, 50}, PlotLegends → Automatic,
   FrameLabel → {"Length of Tubule [nm]", "Number of r=50 nm Holes"},
   PlotLabel → "Sheet Radius", ColorFunction → "SunsetColors",
   ContourShading → None, ContourLabels → True]
Show[
 dp,
 cp]
```

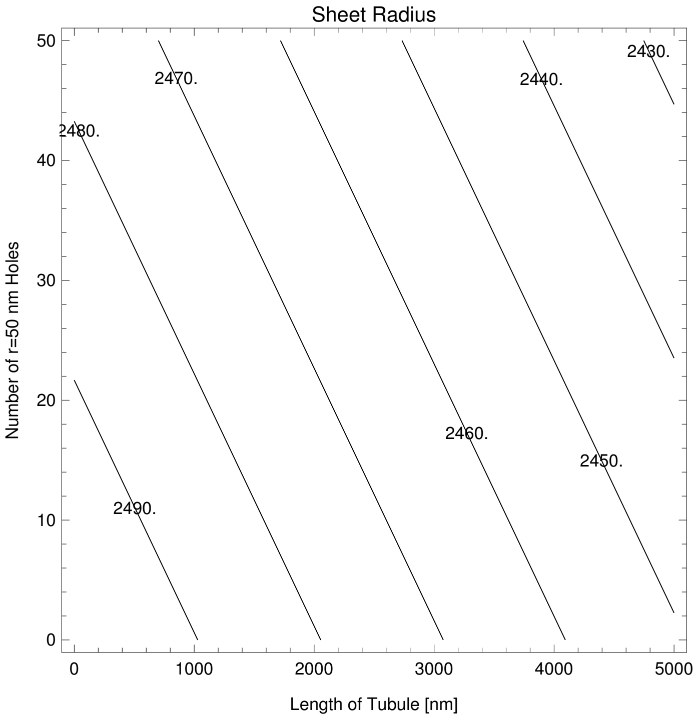

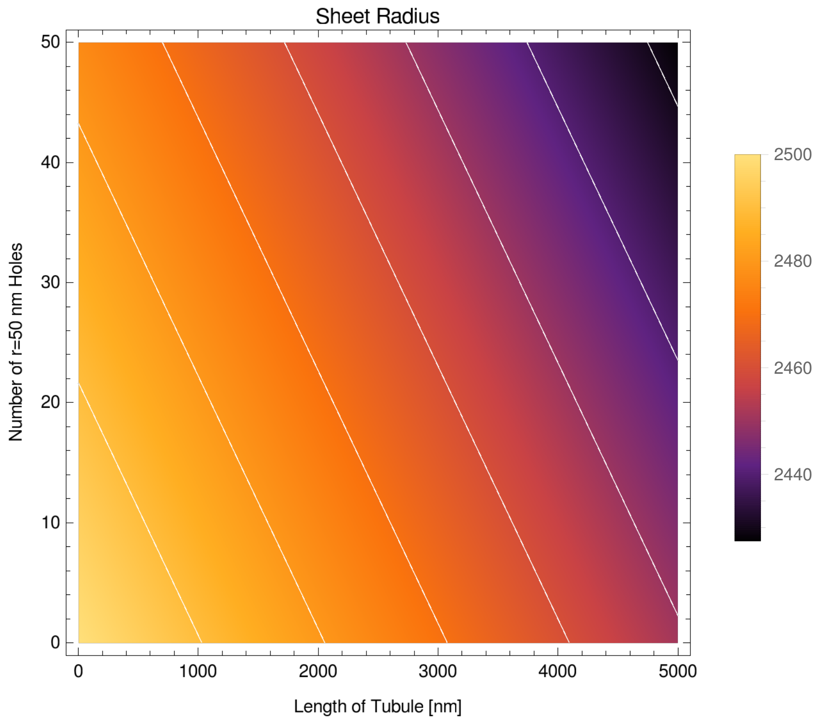

Calculating dimensions for the structure depiction

sheetRadiusPlus[thickness_, tubuleRad_, holeRad_, tubuleLength_, nHoles_]

Note, adding on 25 nm (half the thickness of the sheet), because the fillet in SolidWorks will shave this off, and we defined the unholey sheet radius as the radius of the flat bit.

```
25 + sheetRadiusPlus[50, 50, 50 + (0.5 * 50) , 0, 15] /. constantSurfaceArea → sa50nm
```

```
2526.68
```

Lots of holes, short tubule

```
25 + sheetRadiusPlus[50, 50, 50 + (0.5 * 50) , 218, 48] /.
 constantSurfaceArea → sa50nm
```

```
2528.22
```

Some holes, some tubule

```
25 + sheetRadiusPlus[50, 50, 50 + (0.5 * 50) , 2481, 26] /.
 constantSurfaceArea → sa50nm
```

```
2503.39
```

Few holes, long tubule

```
25 + sheetRadiusPlus[50, 50, 50 + (0.5 * 50) , 4855, 3] /.
  constantSurfaceArea → sa50nm
```

```
2477.08
```

Using fluorescence amplitude to estimate sheet thickness

From (Barentine, et al. 2018, https://doi.org/10.1016/j.bpj.2018.07.028, see supplement), a lumen-labeled tubule profile can be modeled as, where we have removed the normalization factor $\frac{1}{\pi R^2}$:

```
LorConvolvedLumen[x_] =
  ((16 x^2 Γ^2 + (4 R^2 - 4 x^2 + Γ^2)^2)^(1/4) Cos[1/2 ArcTan[4 R^2 - 4 x^2 + Γ^2, 4 x Γ]] -
    ((4 x^2 + Γ^2)^2)^(1/4) Cos[1/2 ArcTan[Γ^2 - 4 x^2, 4 * x * Γ]]) /. {R → r};
```

We can see that in the limit of the PSF FWHM going to zero, the amplitude of this function goes to the diameter of the tubule, as we expect

```
Plot[LorConvolvedLumen[x] /. {r → 50, Γ → 0.1}, {x, -150, 150}]
LorConvolvedLumen[0] /. {r → 50, Γ → 0.1} // N
```

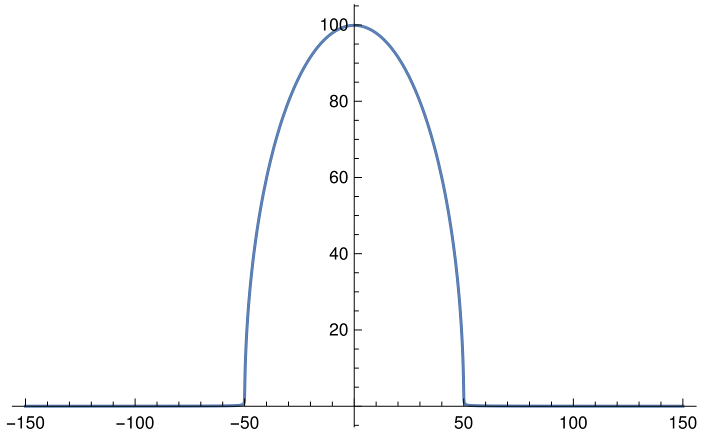


```
99.9
```

We can then plot the brightness of the sheet, and the peak brightness of the tubule, which is decreased because the convolution averages it with surrounding background, rather than other fluorescence signal. An example of this, for the same tubule plotted above but with a larger PSF FWHM is shown

```
Plot[LorConvolvedLumen[x] /. {r → 50, Γ → 50}, {x, -150, 150}]
LorConvolvedLumen[0] /. {r → 50, Γ → 50} // N
```

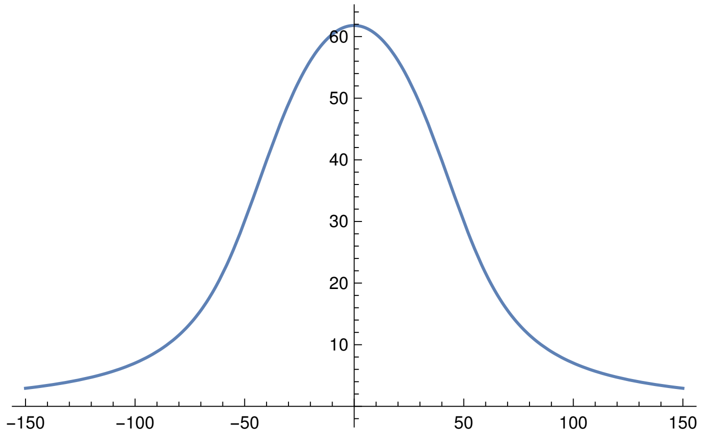

61.8034

```
sheetBrightness[thickness_] := thickness;
peakTubuleBrightness[diameter_] := LorConvolvedLumen[0] /. {r → 0.5 * diameter}
```

```
Plot[{sheetBrightness[thickness], peakTubuleBrightness[thickness] /. {Γ → 50}},
 {thickness, 0, 110}, PlotLegends → {"Sheet", "Tubule"},
 AxesLabel → {"Thickness [nm]", "Peak Fluorescence [A.U.]"},
 ImageSize → Medium, PlotStyle → Thick]
```

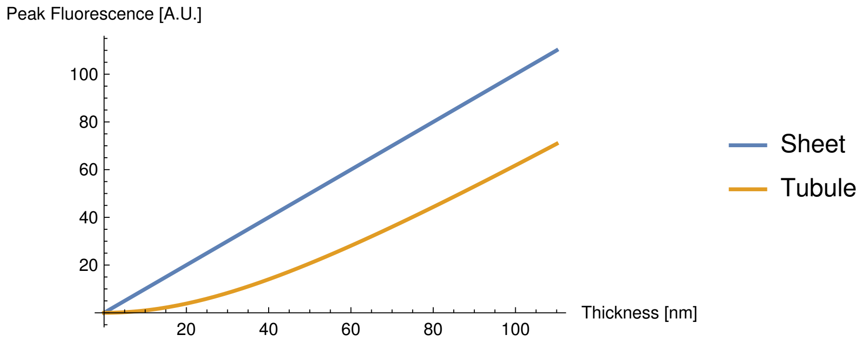

```
DensityPlot[peakTubuleBrightness[diameter] / sheetBrightness[thickness] /. {Γ → 50},
 {diameter, 0, 200}, {thickness, 0, 100}, PlotLegends → Automatic,
 FrameLabel → {"Tubule Diameter [nm]", "Sheet Thickness [nm]"},
 PlotLabel → "Peak Tubule Brightness / Sheet Brightness",
 ColorFunction → "SunsetColors", PlotPoints → 200]
```

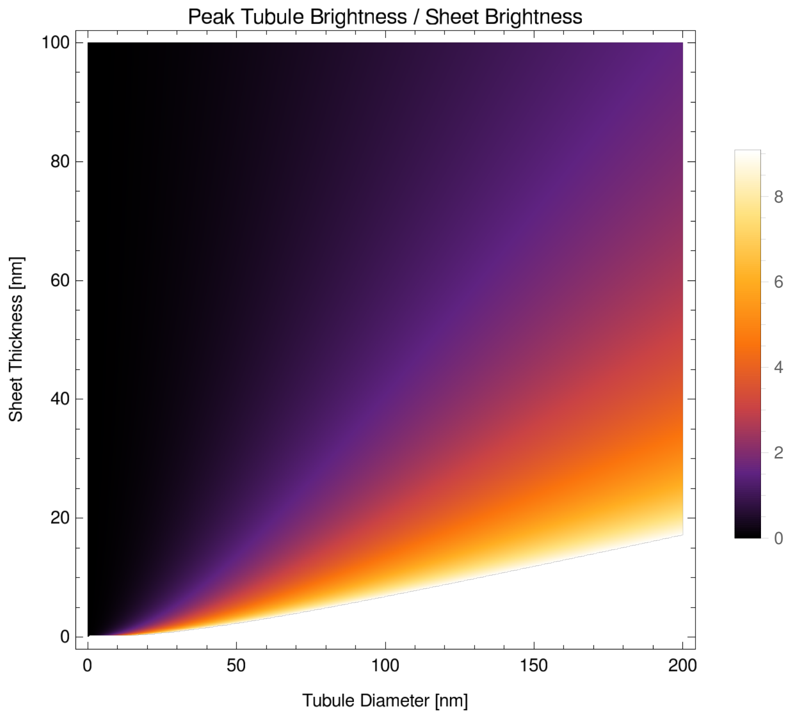

```
DensityPlot[peakTubuleBrightness[diameter] / sheetBrightness[thickness] /. {Γ → 50},
 {diameter, 30, 180}, {thickness, 20, 110}, PlotLegends → Automatic,
 FrameLabel → {"Tubule Diameter [nm]", "Sheet Thickness [nm]"},
 PlotLabel → "Peak Tubule Brightness / Sheet Brightness",
 ColorFunction → "SunsetColors", PlotPoints → 200]
```

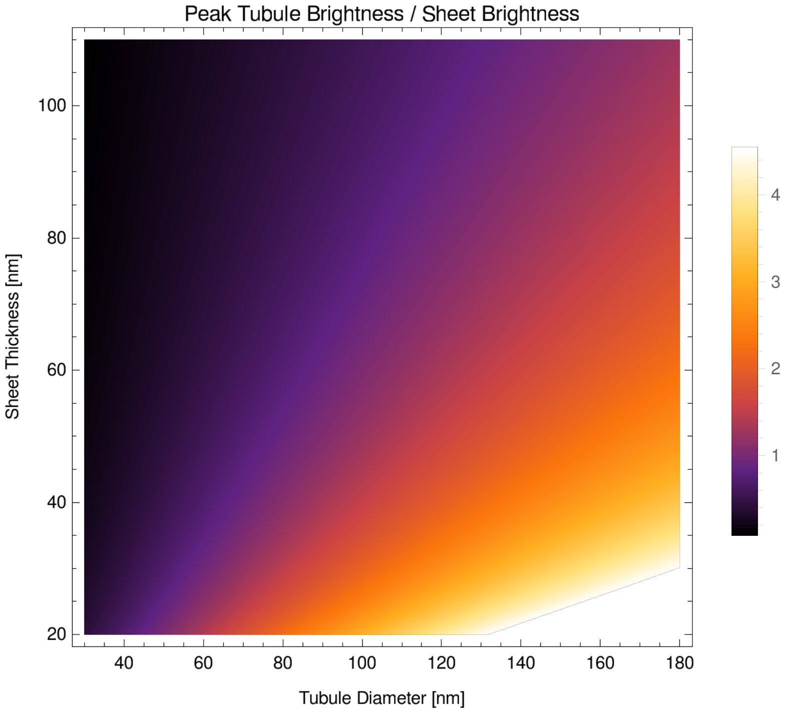

Calculating the fluorescence ratio for a 30 nm thick sheet, 100 nm diameter

```
peakTubuleBrightness[100] / sheetBrightness[30] /. {Γ → 50}
```

```
2.06011
```

Calculating the fluorescence ratio for a 50 nm thick sheet, 100 nm diameter

```
peakTubuleBrightness[100] / sheetBrightness[50] /. {Γ → 50}
```

```
1.23607
```
